# Multi-biological activity evaluation of Sn(П), Zn(П) and Fe(П) complexes based on thiocarbohydrazide schiff bases: synthesis, spectroscopic investigations and fluorescence studies

**DOI:** 10.1038/s41598-025-09239-w

**Published:** 2025-08-28

**Authors:** Abdulsalam Mahdy, Jalal A. Zahra, Randa N. Haddadin, Yusuf Al-Hiari, Violet Kasabri

**Affiliations:** 1 Chemistry Department, School of Science, The University of Jordan, Amman, Jordan; 2 Chemistry Department, Faculty of Education & Science - Rada’a, Albaydha University, Albaydha, Yemen; 3 Department of Pharmaceutics & Pharmaceutical Technology, School of Pharmacy, The University of Jordan, Amman, Jordan; 4 Department of Pharmaceutical Sciences, School of Pharmacy, The University of Jordan, Amman, Jordan; 5 Department of Biopharmaceutics and Clinical Pharmacy, School of Pharmacy, The University of Jordan, Amman, 11942 Jordan

**Keywords:** Thiocarbohydrazide schiff bases, Drug resistance, Metal complexes, Fluorescence turn-on, Antibacterial activity vs. ciprofloxacin, Antifungal activity vs. fluconazole, Selective cytotoxicity vs. cisplatin, Lipopolysaccharide induced inflammation in macrophages vs. indomethacin, DPPH radical scavenging vs. antioxidant ascorbic acid, Cancer, Chemical biology, Developmental biology, Chemistry

## Abstract

Two new Schiff base ligands (L1 and L2) were synthesized by condensing thiocarbohydrazide (TCH) with *o*-anisaldehyde or *p*-anisaldehyde in ethanol. Their mono- and bi-nuclear complexes with Sn(II), Zn(II), and Fe(II) were prepared for potential fluorescence and biological applications. Characterization was performed using FT-IR, NMR, UV-Vis spectroscopy, mass spectrometry, molar conductance, TGA, X-ray diffraction and SEM. XRD results indicated good crystallinity with crystallite sizes of 20–50 nm. Fluorescent intensity of free TCH ligands increased upon complexation with Sn, Zn, and Fe, suggesting their potential as fluorescence chemosensors. The compounds exhibited variable antimicrobial activities against *Staphylococcus aureus*, *Escherichia coli*, and *Candida albicans*, but lower than commercial drugs. L1Fe and L1Zn enhanced L1’s cytotoxicity in four colorectal malignancy cells and L1Zn in skin cancer cells (A375), lung cancer cells (A549), uterine cervix cells (HeLa), and glioblastoma cells (U87). L1Fe showed enhanced activity in mammary adenocarcinoma cells (T47D) and triple-negative breast cancer cells (MDA-MB-231). L2Sn exhibited 70fold increase in L2’s DPPH radical scavenging compared to the antioxidant ascorbic acid. L1Zn and L2Zn complexes outperformed indomethacin in reducing inflammation in RAW macrophages, enhancing the nanomolar efficacy of L1 and L2. These complexes have promising utility in cancer diagnosis, monitoring and highly selective duality of anti-inflammatory/cytotoxicity treatments.

**Supplementary Information:**

The online version contains supplementary material available at 10.1038/s41598-025-09239-w.

## Introduction

Schiff bases (SBs) have a wide range of physico-chemical, catalytic, and structural characteristics, which makes the synthesis and study of several kinds of them and their metal complexes significant^1–7^. Since polydentate SBs and their metal complexes are readily available, many studies have examined their structure-property correlations. SBs are very important in medicine and pharmacy since metal chelates seem to be engaged in various biological activities, including carboxylation, racemization, and transamination^8,9^. Designing new metal-based Schiff bases is a successful tactic to combat the present threat of antioxidant, antibacterial, and anticancer resistance^10–19^. Metal complexes offer an incredibly diverse and reliable technique for the synthesis of better pharmaceutical substances. Indeed, by regulating the metal center oxidation state and choosing the best ligands for each application, it is possible to adjust the chemical properties of these complexes precisely. Therefore, it is not surprising that the issue of creating novel and enhanced metal-based drugs resembles that of creating novel and enhanced ligands for the functional metal element^20,21^. Generally, due to their many uses in industry, medicine, and analytical chemistry, molecules with thione (> C = S) and thiol (> C-SH) groups hold a significant place among organic molecules^22,23^. The functionalization of thiocarbohydrazide (TCH) and its applications in organic synthesis have been the subject of interesting publications in recent years^24^. Additionally, TCHs have garnered significant attention lately because of their antibacterial, antiproliferative, and anticarcinogenic properties^25,26^. These ligands have interesting features, such as structural flexibility and the presence of both soft and hard donor atoms. Numerous structural options for their corresponding metal complexes are made possible by the tautomerism of these ligands and the tendency of oxygen and sulfur donors to function as bridging sites.Additionally, TCHs have been used as chelating ligands to create complexes that contain mono, di, or multi-nuclear, and they are used as building blocks for structures that self-assemble^27^. To the best of our knowledge, there has been relatively a little research on organometal complexes of TCHs, despite several studies regarding their chelating behavior with transition metals^28–31^. A mononuclear organotin complex was created in these publications by coordinating tin with only one portion of the TCH in a doubly deprotonated O, N, S manner. Also, new cis-dioxomolybdenum(VI) complexes with tridentate ONS-donor ligands based on TCHs were synthesized and their antioxidant activity was studied. A novel Schiff base of pentadentate ONSNO-based thiocarbohydrate, and only its dioxomolybdenum (VI) complex was prepared for antioxidant activity^27^. Lastly, a TCH ligand’s binary and ternary metal complexes were synthesized for antibacterial application^32^. However, there are currently very few reports on the biological activity of TCH ligands. In this study, we designed novel bioactive complexes based on TCH ligands containing methoxy groups in *ortho* and *para* positions of the benzene ring. The molecular structure of the targeted ligands and their metal complexes was fully characterized by FTIR, NMR (^1^H, ^13^C), UV, and HRMS. The crystalline nature of the TCH ligands and their metal complexes was investigated using scanning electron microscopy (SEM) and X-ray diffraction (XRD). Our new system’s fluorescence, thermal, and biological properties were studied in detail.

## Experimental

### Materials and methods

Uncorrected melting points of the ligands and their metal complexeswere determined in open capillary tubes on a Gallenkamp electrothermal melting temperature apparatus. ^1^H, ^13^C -NMR spectra were measured on a Bruker Avance-III 500 MHz spectrometer in DMSO-d6 with TMS as an internal standard. The IR spectra were recorded using a Mattson 1000 in the 400–4000 cm^− 1^ wavenumber range. The UV Absorption and fluorescence spectra of the TCH ligands and Sn, Zn, and Fe complexes were recorded using an HP spectrophotometer (Agilent 8453) and an Eclipse Fluorescence Spectrophotometer, respectively. The samples were prepared in DMF solution (10^− 4^ M) at room temperature. The excitation of each the PL samples were 350 nm and the slit widths of excitation and emission measurements was 5 nm. Conductivity measurements were determined on a Lap PH meter inolab^®^ PH 7110 at 21 °C in DMF (10^− 3^M). The thermal behavior of the TCH ligands and their complexes was investigated by TGA analysis using a Netzsch STA 409 PG/PC thermal analyzer (Selb, Bavaria, Germany). The SEM images were recorded in a Hitachi SEM analyzer, and XRD was recorded on a RigakuDmax X-ray diffractometer with CuKa radiation (k = 1.5404 A˚). All microbiological media used were from Liofilchem-Italy. Ciprofloxacin HCl and Fluconazolewere procured from Thermo Fisher Scientific. All chemicals from Sigma were of analytical grade. Human PC3 prostate cancer cell line (ATCC^®^ CRL-1435), A375 human skin cancer cell line (ATCC^®^ CRL-1619), A549 lung cancer cell line (ATCC^®^ CCL-185), HeLa uterine cervix adenocarcinoma cell line (ATCC^®^ CRM-CCL-2), U87 glioblastoma (ATCC^®^ HTB-14 ), Breast cancer cell line MCF7 (ATCC^®^ HTB-22), T47D (ATCC^®^ HTB-133), MDA-MB-231 (ATCC^®^HTB-26), MDA-MB-453 (ATCC^®^HTB-131), BT-549 (ATCC^®^HTB-122), PANC1 pancreatic cell line (ATCC^®^ CRL-1469), and colorectal cancer cell lines namely; HT-29 (ATCC^®^ HTB-38), HCT116 (ATCC^®^ CCL-247), SW480 (ATCC^®^ CCL-228), SW620 (ATCC^®^ CCL-227) and CACO2 (ATCC^®^ HTB-37) and RAW mouse 264.7 macrophage cell line (ATCC^®^ TIB-71) cell lines were procured as well.

### Preparation of 1, 5-bis(2-methoxyanisaldehyde) thiocarbohydrazine(L1)

TCH ligands were prepared according to the procedure previously reported^33,34^. To a 100 -ml -bottomed flask, 10 mmol (1.5 g) of *o*-anisaldehyde and 5 mmol (0.5 g) of TCH were added with 30 ml of absolute EtOH and 5 ml of glacial acetic acid with stirring at 70 °C. After 5 h, the reaction mixture was cooled and kept overnight. The solid precipitate was filtered and washed with water, EtOH, and petroleum ether. The beige crystal ligand was obtained from ethanol and then dried in a vacuum oven, yielding 1.4 g (95%). FT-IR (ν cm^− 1^): 3453(OH /H_2_O), 3156(NH), 2837(C-H methoxy group), 1628, 1606((C = N-N = C)), 1524(C = C), 1393(NH-C = S) and 1450(C-N), 1257(C-O methoxy), 755 (C = S). ^1^H-NMR (500 Hz, DMSO, δ ppm):12.37(OH/H_2_O), 11.6 and 11.7 (s 2 H, N-H), 8.92 and 8.52 (2 H, HC = N), 7.9–6.9 (8 H, C-H _aromatic_), 3.85(6 H, OCH_3_). ^13^C-NMR (500 Hz, DMSO, δ ppm): 175(1 C, C = S), 158(2 C, C-O-C-), 144 and 140(2 C, N = C-H), 131 − 110(10 C, C_aromatic_), 55.5(2 C, O-CH_3_). HRMS (ESI): m/z [M + K]^+^calcd for C_17_H_18_KN_4_O_2_S: 381.08; found: 381.078. λ_max_(nm): 255 π-π*(aromatic rings), 344 n-π*(-C = N-), 353 n-π*(-C = S).

### Preparation of 1, 5-bis (4-methoxyanisaldehyde) thiocarbohydrazine (L2)

To a 100–ml-bottomed flask, 10 mmol (1.5 g) of *p-*anisaldehyde and 5 mmol (0.5 g) of TCH were added with 30 ml of absolute EtOH and 5 ml of glacial acetic acid with stirring at 70 °C. After 10 h, the reaction mixture was cooled and kept overnight. The solid precipitate was filtered and washed with water, EtOH, and petroleum ether. The white crystal ligand was obtained from ethanol and then dried in a vacuum oven, yielding 1.3 g (86.7%). FT-IR (ν cm^− 1^): 3453(OH/H2O), 3170(NH), 2849(C-H methoxy group), 1628, 1609((C = N-N = C), 1546, 1500(C = C), 1393 (NH-C = S), 1256(C-O methoxy), 833(C = S). ^1^H-NMR (500 Hz, DMSO, δ ppm): 11.45 and 11.55 (s 2 H, N-H), 8.54 and 8.09 (2 H, HC = N), 7.95-7.0 (8 H, C-H aromatic), 3.75(6 H, OCH_3_). ^13^C-NMR (500 Hz, DMSO, δ ppm): 175(1 C, C = S), 161(2 C, C-O-C-), 149 and 143(2 C, N = C-H), 130 − 115(10 C, C aromatic), 55.50(2 C, O-CH_3_). HRMS (ESI): m/z [M + K]^+^calcd for C_17_H_18_KN_4_O_2_S: 381.08; found: 381.078. λ_max_ (nm): 263 π-π*(aromatic rings), 338 n-π*(-C = N-), 359 n-π*(-C = S).

### Synthesis of TCHs metal complexes (Sn, zn, and Fe)

A hot methanolic solution (20 mL) of the TCH ligands (L1 or L2) was drop-wise added to a hot methanolic solution (10 mL) of metal (II) chloride salts (Sn, Fe, and Zn) with a molar ratio of 1:2 (ligand: metal) in the case of the L1 complexes and 2:1 in the case of the L2 complexes. After 5 h, the solid precipitate of Sn(II), Fe(II), and Zn(II) TCH ligand complexes, highly colored products, were obtained and subsequently filtered out, washed with methanol, and then dried for 12 h in air.

Spectral data of L1Sn: Yellow solid crystal, yield: 92%; M.P.: 195–200 °C. FT-IR (ν cm^− 1^): 3490, 3203, 1643, 1385, 1250, 742. ^1^H-NMR (500 MHz, DMSO-d_6,_(Figure S2) δ: 12.45 (s, 1H, OH), 11.6 and 11.7 (s 2 H, N-H and N-H), 8.69(s, 1H, HC = N), 8.54(t, 1H, HC = N), 7.90–6.90(m, 8 H, Ar-H), 3.75(t, 6 H, O-CH_3_). λ_max_ (nm): 255 π → π*, 344 n → π*, 353 n → π*.HRMS (ESI): m/z [M + Na]^+^calcd for C_17_H_22_Cl_3_N_4_O_4_SSn_2_: 745.85; found: 745.81.

Spectral data of L1Zn: Ivory solid, yield, 75%, M.P.: 260–265 °C. FT-IR (ν cm^− 1^): 3490, 3203, 1636,1560, 1385, 1250.9, 752. ^1^H-NMR (500 MHz, DMSO-d6, Figure S3) δ: 12.45 (s, 1H, OH), 11.24 and 11.33 (s 2 H, N-H and N-H), 8.82(s, 1H, HC = N), 8.41(t, 1H, HC = N), 7.80–6.70(m, 8 H, Ar-H), 3.70(t, 6 H, O-CH_3_). λmax (nm): 262 π → π*, 335 n → π*, 363 n → π*, 394.HRMS (ESI): m/z [M + K]^+^calcd for C_17_H_16_Cl_4_N_4_O_2_SZn_2_: 648.80; found:648.79(Figure S4).

Spectral data of L1Fe: brown solid, yield, 75%, M.P.:150–155 °C. FT-IR (ν cm^− 1^): 3452, 3197, 1635, 1573, 1380, 1241, 1087, 738. ^1^H-NMR (500 MHz, DMSO-d6Figure S5) δ: 12.30 (s, 1H, OH/H_2_O), 11.50 (s 1H, N-H), 8.89(s, ^1^H, HC = N), 8.40(t, 1H, HC = N), 7.90–6.90(m, 8 H, Ar-H), 3.75 (t, 6 H, O-CH_3_). λmax (nm): 266 π → π*, 321 n → π*, 336 n → π*.HRMS (ESI): m/z [M + Na]^+^calcd for C_17_H_23_ClFeN_4_O_4_S: 492.17; found: 493.03(Figure S6).

Spectral data of L2Sn: yellow solid, yield, 75%, M.P.: 212–215 °C. FT-IR (ν cm^− 1^): 3490, 3203, 1641, 1385, 1250.9, 833, 506. ^1^H-NMR (500 MHz, DMSO-d6, Figure S7) δ: 12.30 (s, 1H, OH), 11.60 and 11.40 (s 2 H, N-H and N-H), 8.42(s, 1H, HC = N), 8.20(t, ^1^H, HC = N), 8.10–6.90(m, 8 H, Ar-H), 3.75(t, 6 H, O-CH_3_).λmax (nm): 253 π-π*, 319 n-π*, 390 n-π*, 470.HRMS (ESI): m/z [M + Na]^+^calcd for C_17_H_21_ClN_4_O_4_SSn: 862.12; found: 865.48(Figure S8).

Spectral data of L2Zn: Ivory solid, yield, 75%, M.P.: 146–150 °C. FT-IR (ν cm^− 1^): 3490, 3203, 1638, 1385, 1250.9, 830, 515. ^1^H-NMR (500 MHz, DMSO-d6, Figure S9) δ: 12.30 (s, 1H, OH), 11.20 and 11.00 (s 2 H, N-H and N-H), 8.60(s, 1H, HC = N), 8.07(t, 1H, HC = N), 8.10–6.90(m, 8 H, Ar-H), 3.75(t, 6 H, O-CH_3_).λmax (nm): 260 π-π*, 323 n-π*, 360 n-π*, 384, 401.HRMS (ESI): m/z [M + 1]^+^calcd forC_34_H_34_N_8_O_4_S_2_Zn: 746.14; found: 746.15(Figure S10).

Spectral data of L2Fe: brown solid, yield, 75%, M.P.: 162–165 °C. FT-IR (ν cm-^1^): 3454, 3280, 1638, 1396, 1269, 1176, 1112, 1035, 846, 515, 814. ^1^H-NMR (500 MHz, DMSO-d6,Figure S11) δ: 12.30 (s, ^1^H, OH/H_2_O), 8.60(s, ^1^H, HC = N), 8.10–6.90(m, 8 H, Ar-H), 3.75(t, 6 H, O-CH_3_). λmax (nm): 282 π-π*, 347 n-π*, 361 n-π*.). HRMS (ESI): m/z [M + Na]^+^calcd for C_34_H_36_FeN_8_O_5_S_2_: 796.12; found: 796.11(Figure S12).

### Minimum inhibitory concentration determination (MIC)

The compounds were tested against Gram-positive bacteria (*Staphylococcus aureus* ATCC 6538), Gram-negative bacteria (*Escherichia coli* ATCC 8739) and yeast (*Candida albicans*ATCC10231). Due to poor water solubility, the compounds were dissolved in dimethyl sulfoxide (DMSO) to achieve a 1000 µg/mL concentration. For bacterial strains, the EUCAST (2025) method was adapted^35^. A few colonies of each bacterial culture previously grown on Mueller-Hinton agar for 24–48 h were suspended in normal saline and adjusted to 0.5 McFarland (1.5 × 10^8^ CFU/mL). A 1/10 dilution was prepared in Mueller-Hinton broth (MHB) for the inoculation. In the wells of a 96-well flat-bottom microtiter plate, double serial dilutions of each compound were prepared in MHB. Each well was inoculated with the bacteria to achieve ca. 5 × 10^5^ CFU/mL, and the plates were incubated at 37 °C overnight. Positive control (bacteria in culture medium only) and negative control (culture medium) were prepared. Each compound was tested in duplicate. After incubation, the plates were inspected visually for growth inhibition. To test the antimicrobial activity of DMSO, double serial dilutions of DMSO in broth medium were prepared and processed as the samples, but without the addition of the compounds. The antibacterial drug ciprofloxacin was used as a reference for comparison.

For *Candida albicans*, the same procedure applied to bacteria was used, except after inoculation, the plates were incubated at 35 °C for 24 h, and after incubation, the absorbance was measured at 530 nm using a plate reader^36^. If the positive control wells showed absorbance ≤ 0.2, the plates were further incubated for another 24 h, and the absorbance was measured again for all the wells. The absorbance of the wells was corrected by subtracting the absorbance of the blank. The MIC was considered the minimum concentration, which gave rise to an inhibition of growth of ≥ 50% of the positive controls. The antifungal drug fluconazole was used as a reference for comparison^36^. Since DMSO resulted in growth inhibition at concentrations of 12.5% and above for the bacteria and the yeast tested, MIC was considered the minimum concentration of the compound that results in inhibition of the growth where DMSO concentration is 6.25% or below, therefore, the highest concentration of the drugs that can be achieved without interference in activity from DMSO is 62.5 µg/mL.

### Viability assays for antiproliferative capacities of test compounds: sulforhodamine B (SRB) assay

All malignancy cell lines were cultured in high glucose DMEM (Bio Whittaker, Verviers, Belgium) containing 10% FBS, 4-(2-hydroxyethyl)-1-piperazineethanesulfonic acid (HEPES) Buffer (10 mM), L-glutamine (2 mM), gentamicin (50 µg/mL), penicillin (100 U/mL), and streptomycin sulfate (100 mg/mL) from Sigma (St. Luis, MO, USA) whereas Sulforhodamine B was from Santa Cruz Biotechnology (Inc. Texas, USA). Surviving cancer cell lines provide numerous advantages; they provide a pure and continuous supply of cells, thus overcoming any ethical barriers concerning human tissue usage, and are cost-effective. For cytotoxicity screening, adherent monolayers of all procured malignancies’ cell lines were examined. Periodontal ligament fibroblasts (PDL) were used to determine selective cytotoxicity. The cytotoxicity measurements were determined using Sulforhodamine B (SRB; Santa Cruz Biotechnology, Inc., Texas, USA) as a colorimetric assay for cytotoxicity screening (using Spectro Scan 80D UV-VIS spectrophotometer (Sedico Ltd., Nicosia, Cyprus)^37^. Cells were incubated with compounds or reference agents at different concentrations (3.125-200 µM). As a robust and classical antineoplastic apoptogenic reference agent, cisplatin was recruited for comparison purposes^38^. The mechanism of reduction of cell viability was adopted so that dose–response curves were plotted and values were expressed as percentage of control optical density, and IC_50_ values, 50% inhibitory concentration, were estimated by regression analysis. Triplicate/quadriplate assay approach was performed, and the calculated anti-proliferative activities were reported as mean IC_50_ values of tested ± SD (*n* = 3–4).

### Antiinflammatory (nitrite) determination in vitro

RAW 264.7 mouse macrophage cell line (ATCC^®^ TIB-71) were cultured in high glucose DMEM supplemented with 10% (FBS), penicillin (100 U/mL), streptomycin (100 µg/mL), and L-glutamate (100 µg/mL) in a 37 °C humidified atmosphere with 95% air and 5% CO_2_. Confluent macrophages (2 × 10^5^/well) were incubated with inflammation-priming lipopolysaccharide (LPS; Sigma, St. Luis, MO, USA) with either indomethacin as the reference agent^39^ or treatment compounds at different concentrations (1.563-200 µM). Griess reagent was mixed with aliquots of cell culture media and incubated at R.T. for 10 min. Absorbance at 550 nm was determined using a microplate reader (Spectro Scan 80D UV-VIS spectrophotometer (Sedico Ltd., Nicosia, Cyprus). The nitrite concentration was determined by comparison with a sodium nitrite standard curve. SRB cytotoxicity protocol was performed for evaluation of the effect of the studied test compounds on RAW 264.7 viability^40^.

### DPPH free radical scavenger assay

This method depends on the reduction of the radicals, resulting in a color change from oxidized purple to reduced yellow. Principally, Diphenyl-2-picryl-hydrazyl (DPPH) undergoes reduction in methanol (MeOH) solution, in the presence of a hydrogen-donating compound, due to the formation of the non-radical form DPPH-H. This color change can be quantitatively measured using a 515–520 nm spectrophotometer. In contrast to other radical scavenging assays, a DPPH radical is stable and can provide reproducible spectroscopic values^41,42^. A DPPH solution (0.2 mM) was diluted with MeOH and then mixed with treatment compounds as well as ascorbic acid in a concentration ratio of 1:1 using a 96-well plate (so that a final concentration range 6.25–200 µM was obtained for test agents); the treated solution was incubated one hour isolated from light. Finally, a change in absorbance at 517 nm wavelength was measured using a microplate reader (Bio-Tek Instrument, USA). Ascorbic acid was the robust and classical standard radical scavenging reference agent for comparison purposes^43,44^. The calculation of the DPPH radical scavenging activity inhibition was determined by the following equation, where A represents photometric absorbance: in % = (A control – A sample) / A control x 100%)^45^.

### Statistical analysis

The values were presented as mean ± SD of 3–4 independent experiments, determined using GraphPad Prism software [version 5.0 for Windows; GraphPad software, San Diego, CA, USA]. Moreover, for comparative studies, ANOVA with subsequent Dunnette’s post hoc test was implemented and P value < 0.05 was set as the significance cutoff level.

## Results and discussion

TCH ligands (L1 and L2) were prepared from the condensation reaction of TCH and *para* or *ortho-*anisaldehyde in ethanol as solvent. The prepared TCH ligands were obtained with a high yield, and they have good solubility in polar aprotic solvents, DMF, DMSO, and are also partially soluble in chloroform and acetone. The IR, NMR (^1^H, ^13^C), UV, and HRMS results have all provided satisfactory characterizations for the molecular structures of the TCH ligands. For example, in the ^1^H-NMR spectra, the disappearance of a broad signal between 4.5 and 5.0 ppm that is attributed to the NH_2_ group in the TCH core and the appearance of a clear signal at 8.5 and 8.9 ppm for L1 and 8.0 & 8.5 ppm for L2, which correspond to imine groups (HC = N), supports the formation of TCH Schiff bases. In addition, HRMS (ESI) for the C17H18K1N4O2S1 (L1 and L2): m/z [M + K]^+^calcd: 381.08; found: 381.078. Also, the prepared metal complexes showed good stability and crystallinty property. The melting points for the TCH ligands are in the range of 180–190 °C (Table S1), while their complexes, L1Sn, L2Sn, and L1Zn, showed higher melting point values, 200, 215, and 265 °C, respectively, indicating that these complexes have higher thermal stability. Both complexes showed low molar conductance values between 3.6 and 76 µs/cm in DMF (10^− 3^M) (cf. Table S3), indicating that they are of a non-electrolyte nature^46^. The thermogravimetric, spectroscopy, analytical data, and molar conductance allowed us to predict the probable structure of the synthesized complexes.

### Characterizations of TCH ligands and their metal complexes

#### FTIR spectra

**Fig. 1:**
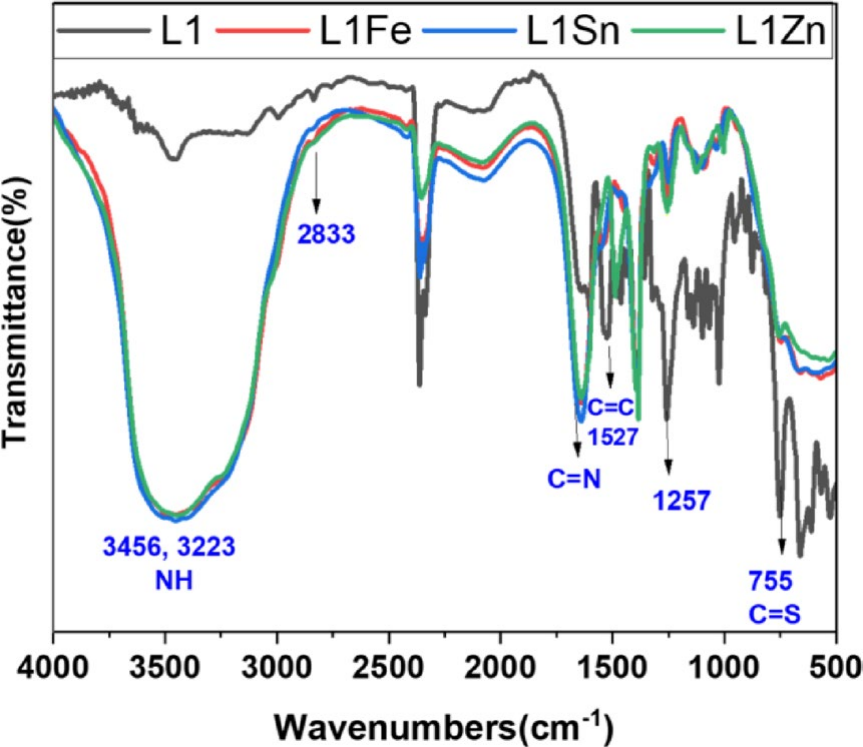
FTIR of L1 and its metal complexes.

**Fig. 2:**
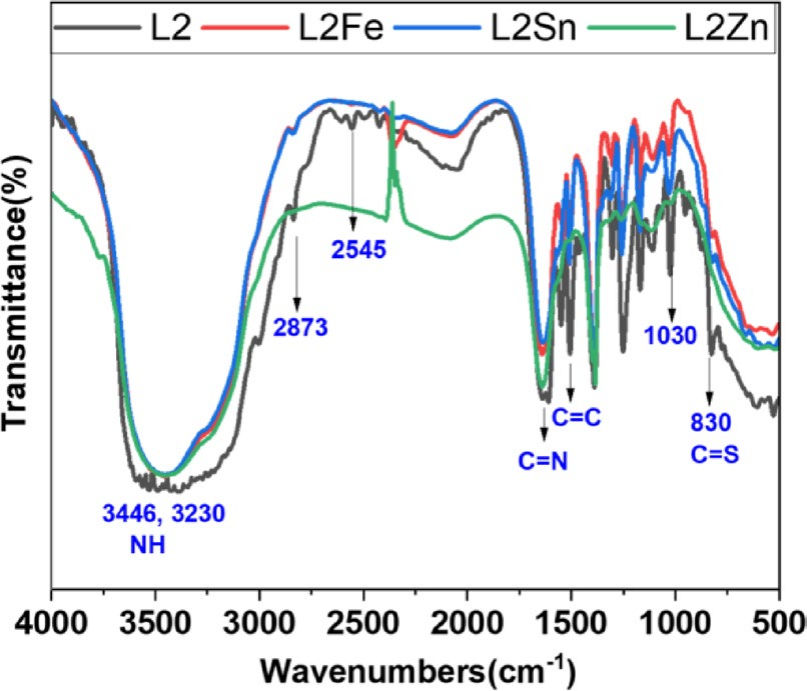
FTIR of L2 and its metal complexes.

The structures of TCH ligands and their metal complexes were confirmed by FTIR analysis, as shown in Figs. 1 and 2. The results support the formation of thione and thiol forms through the coordination of TCH metal complexes. The two bands centered at 1606 and 1628 cm⁻¹ were observed in the spectra of the L1 and L2, which are ascribed to the formation of two azomethine groups(C = N)^47^. These bands are shifted to one broad band at higher frequencies of 1643, 1636, and 1635 cm⁻¹ in the L1 complexes and also to 1641, 1638, and 1638 cm⁻¹ in the L2 complexes spectra. This result indicates that nitrogen atoms of N = C are participating in bonding with metal ions of the complexes^48^. Also, the NH of TCH core was observed as two broad bands at frequencies between 3255 and 3129 cm⁻¹, indicating the presence of the two NH groups in each L1 and L2 metal complexes, revealing to the thione form, a similar result was previously reported^49^. Except in the case of the L2Fe and L2Zn complexes, the coordination with metal ions was in the thiol form, consistent with their proposed structures. The downfield shift of the C = S of the ligands on complexation was observed in each complex, indicating that the sulfur atom participates in bonding with metal ions. Also, in spectra of the L1 metal complexes, the sulfur atom is involved in bonding, which is confirmed by the shifting of (C = S) bond, without the occurrence thiol tautomer. IR spectra of L1 and L2 also showed a strong band at 1257 cm-and a weak band at 2828 cm ^− 1,^ which are assigned to vibration of C-O of R-O-CH_3_ methoxy group and its C-H vibration, respectively. No change in their position was observed in each L2 complex, indicating that the oxygen of the methoxy group does not participate in complexation, except in the case of the L1Zn and L1Sn complexes, where these bands are shifted to higher energy, indicating participation of oxygen methoxy group in coordination^50^. Furthermore, the presence of coordinated water molecules is shown by broad bands appearing at 3389–3415 cm^− 1^ in complexes, which may be attributed to the O–H stretching vibration. In addition, during the complexation, two new bands in the TCH metal complex spectra were observed between 480 and 680 cm⁻¹, which are attributed to M-N and M-S bonds. Thus, from these results and those listed in Table S2, it was concluded that the L1 and L2 act as a didentate (NS or NO), as well as tridentate (NSO) donor sites.

#### NMR spectra

**Fig. 3:**
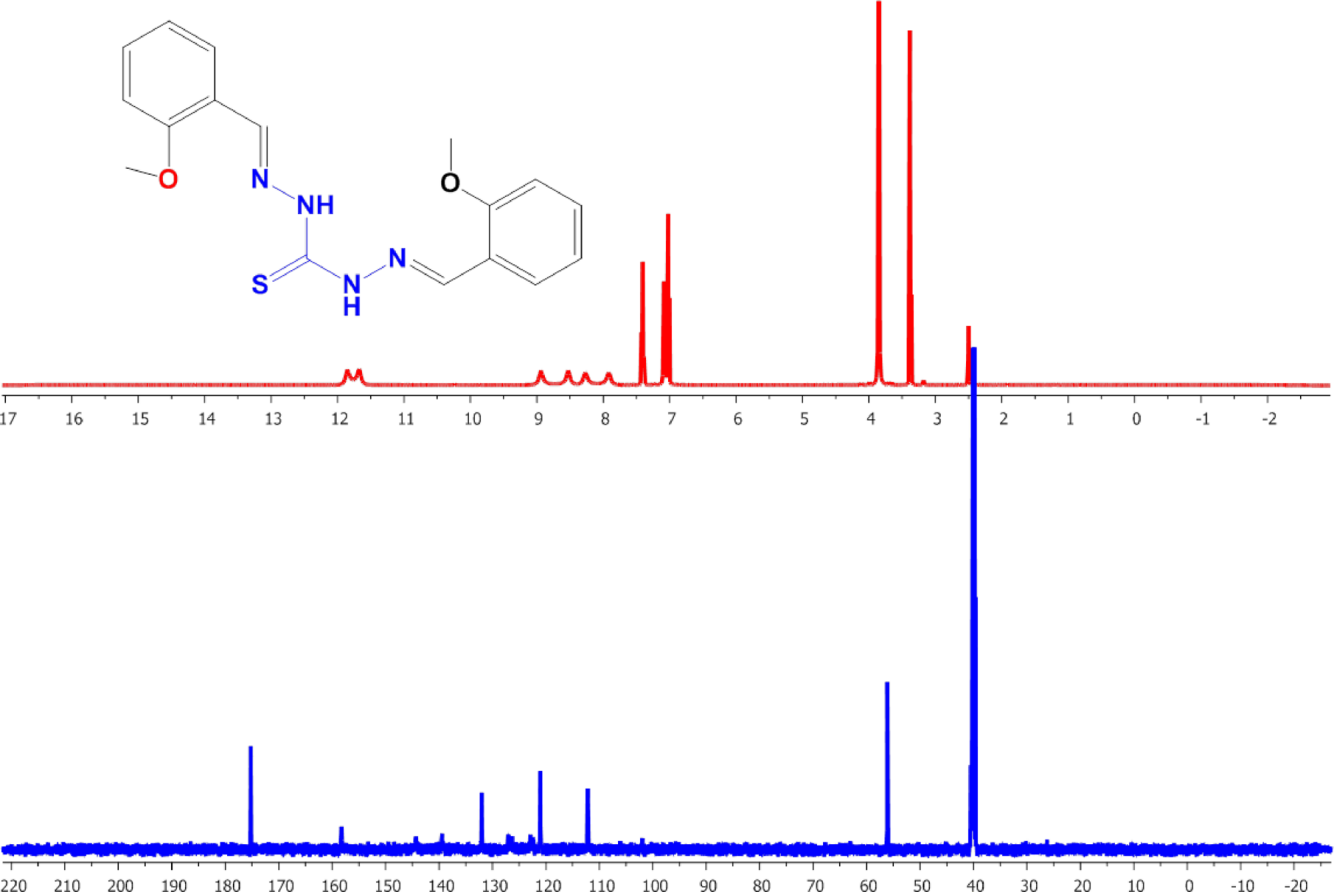
^1^H and ^13^C-NMR spectra of L1.

**Fig. 4:**
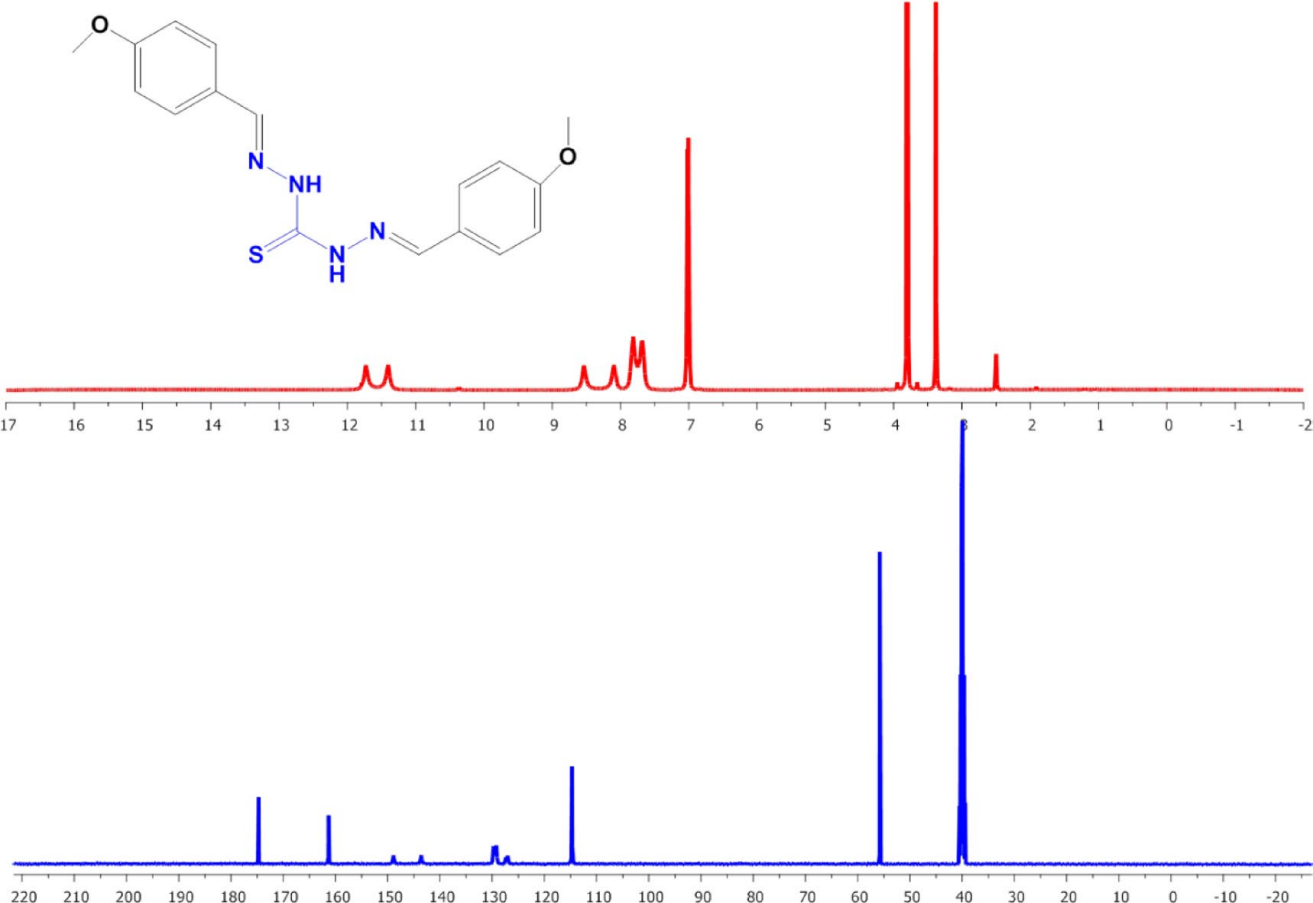
^1^H, ^13^C-NMR spectra of L2.

The structure of the TCH ligands and their complexes was also examined using NMR (^1^H, ^13^C) spectra. The ^1^H-NMR spectra of free L1 and L2 ligands (Figs. 3 and 4) exhibited two signals for the NH- groups at highly downfield of 11.6, 11.7 ppm of L1 spectra and at 11.55, 11.45 ppm of L2 spectra, supporting the presence of intramolecular hydrogen bonding^26^. One of them upfield shifted, was observed in NMR spectra of L2Zn and L2F2 complexes (Figures S2 to S6), supporting the occurrence of thiol form, consistent with their proposed structures^51^. In addition, ^1^H-NMR spectra of free L1 ligand showed two signalscentered at 8.5 and 8.9 ppm, which are attributed to CH = N protons, as were observed at 8, 8.5 ppm for free L2. On complexation, satellites appeared for one of them due to its coordination with metals, indicating that one of N = C azomethine nitrogen is involved in the formation of the complexes. Except in the case of L1Zn and L1Sn complexes, it was observed that one of these signals was satellite and another was upfield shifted, supporting the formation of binuclear complexes. On complexation, new signals appeared between 12 and 13 ppm, indicatingthe presence of coordinated or non-coordinated water molecules.

#### Mass spectra

**Fig. 5:**
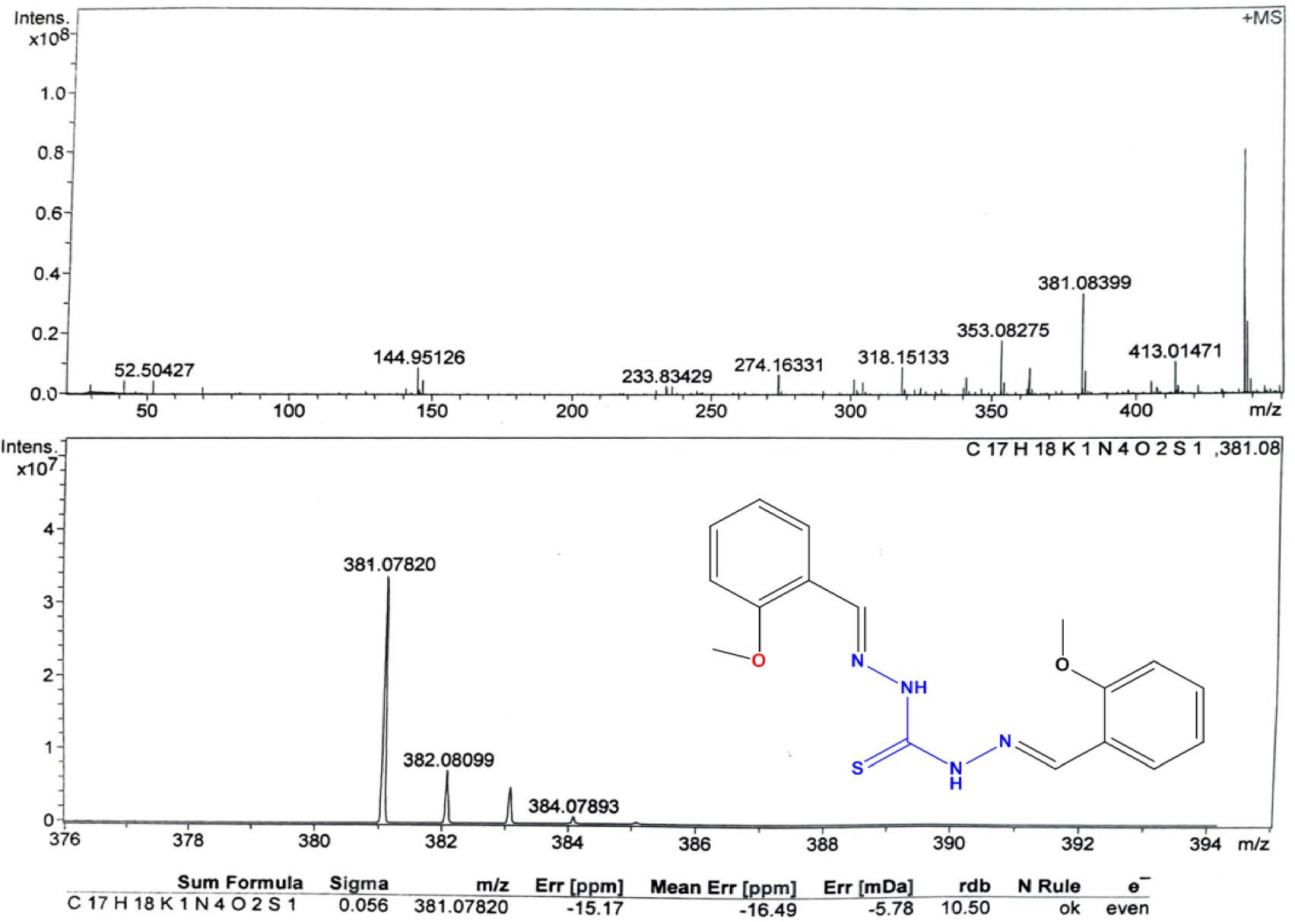
Mass spectrum of L1.

**Fig. 6:**
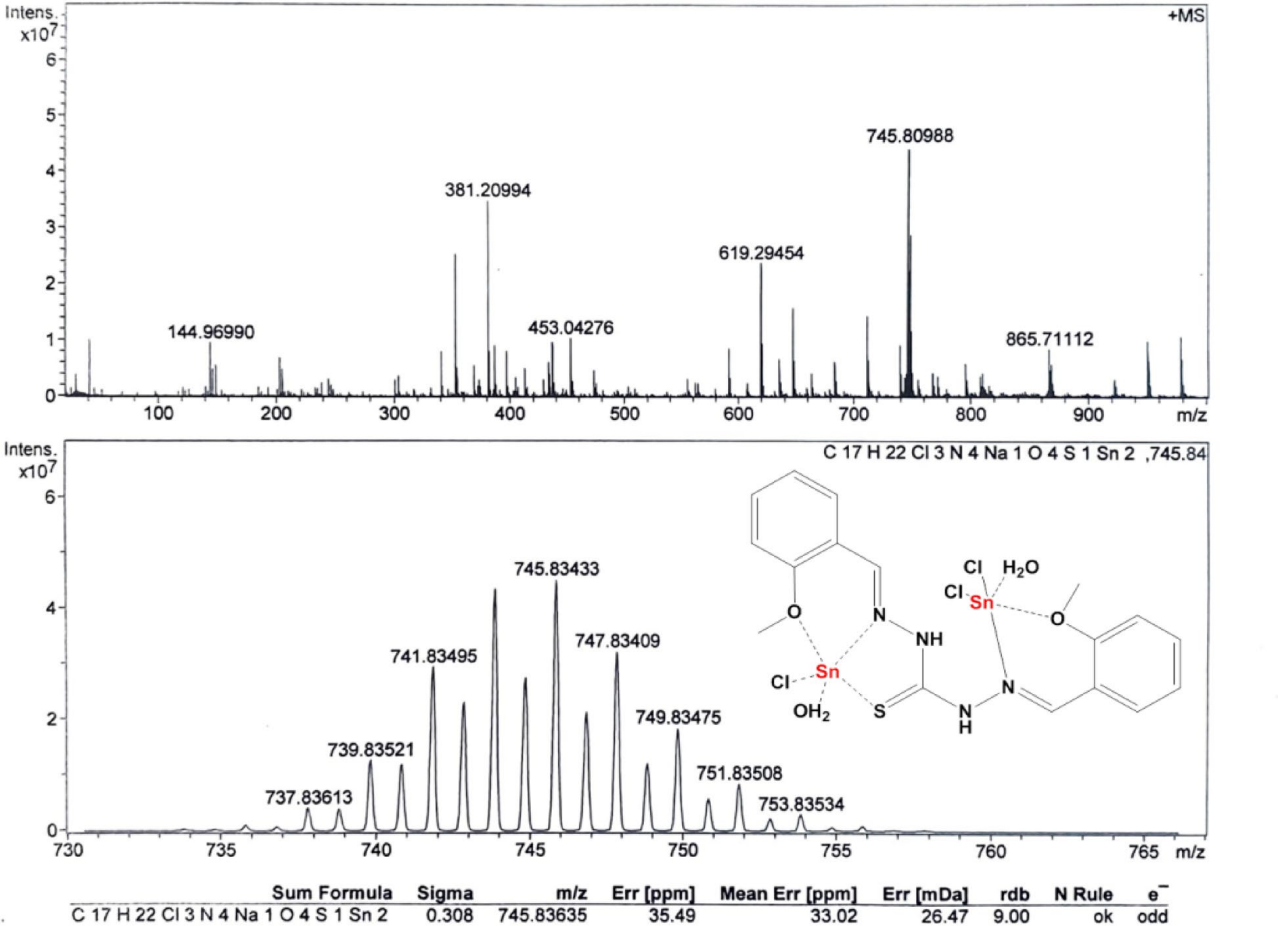
Mass spectrum of L1Sn complex.

In molecular chemistry, mass spectrometry is commonly recognized as a powerful method for determining the structure of molecules. The employment of this method has demonstrated a significant efficacy in coordination chemistry, particularly in identifying the main molecular ion peaks found in the synthesized Schiff base complexes. The mass analysis was conducted using the mass spectrometry technique known as high-resolution mass spectrometry (HRMS). The mass-to-charge ratio (m/z) peak value of the TCH ligands was found at 381.07 (Fig. 5), which is in strong agreement with the calculated value (381.08) and with their chemical formula. Furthermore, the mass spectra of each complex were obtained and summarized in Table S1; for example, the mass-to-charge ratio (m/z) peak value of the L1Sn complex was found at 745.81 (calculated, 745.84; M + Na), most consistent with its proposed molecular formula (Fig. 6). The complexes formation was also confirmed when the parent TCH ligand ion peak showed up in each complex’s mass spectrum at 381 amu. However, the mass spectra of each the complexes are shown in the supplementary file (Figures S7 to S11).

#### Electronic absorption spectra and fluorescence spectra

**Fig. 7:**
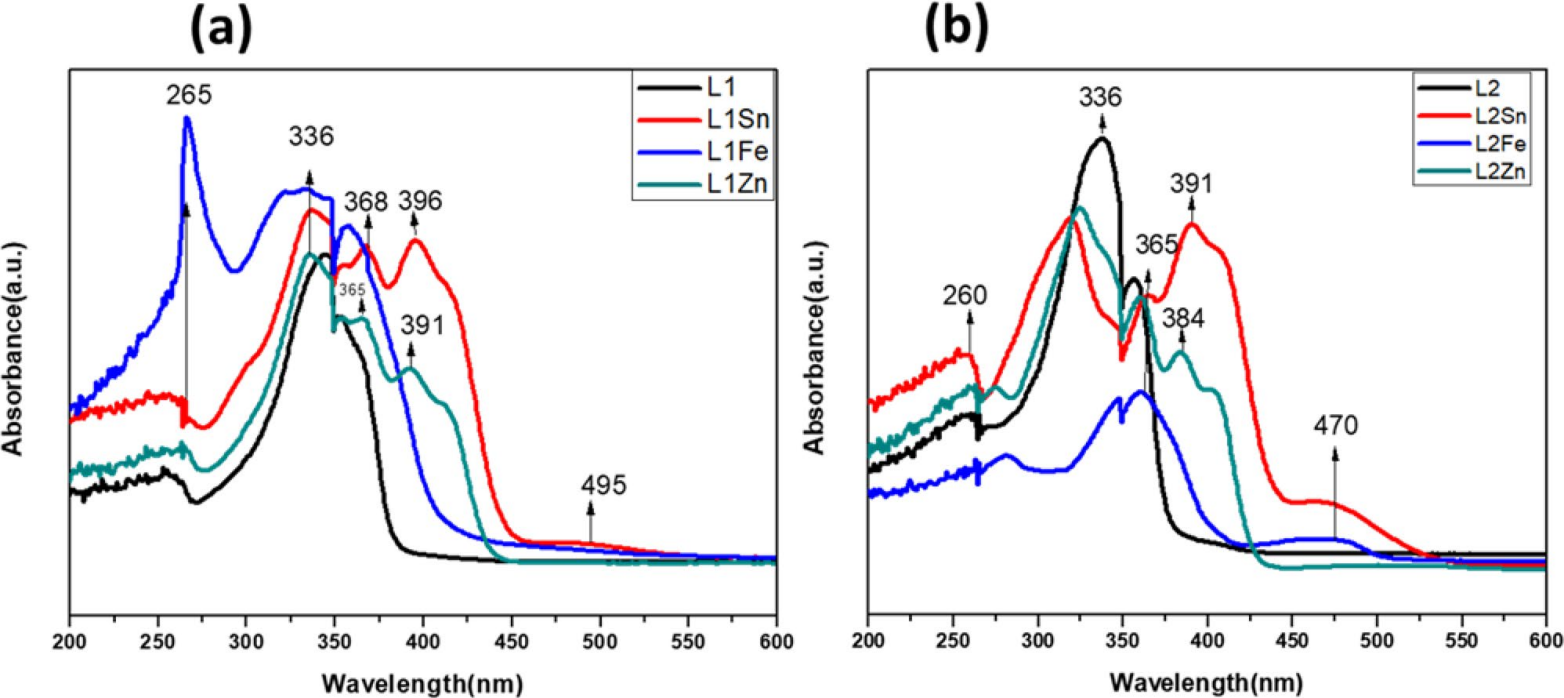
UV spectra of (**a**) L1 and (**b**) L2 ligands and their metal complexes.

**Fig. 8:**
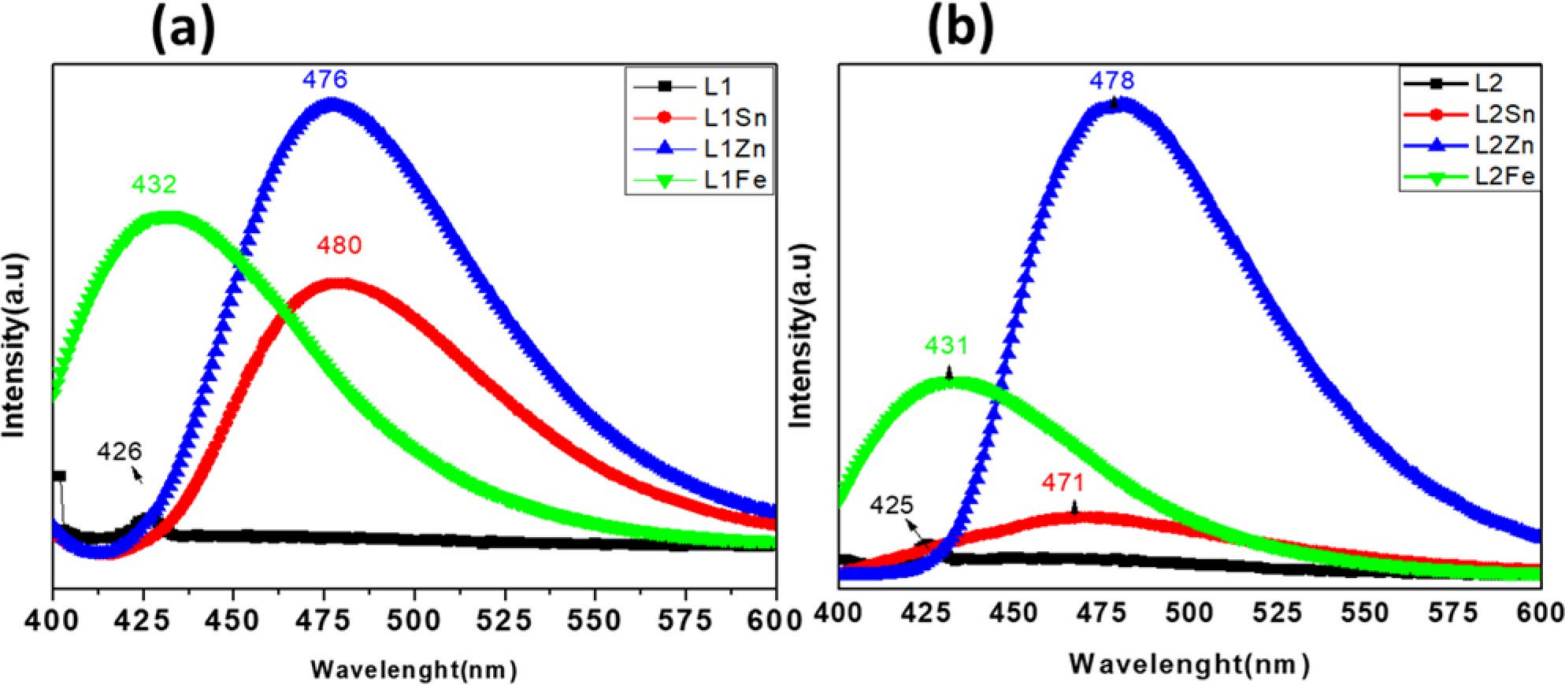
Fluorescence spectra of (**a**) L1 and (**b**) L2 ligands and their metal complexes.

UV–Vis spectral measurements of the TCH ligands and their metal complexes were investigated at room temperature in DMF solvent (5 × 10⁻⁵ M) in the range 200–800 nm, as shown in Fig. 7(a) and (b), and the tentative assignments are summarized in Table S3. Two broad absorption bands and one shoulder were observed at λ_max_ of 277, 344, and 353 nm for the free L1 ligand, and are shifted to higher and lower frequencies in the metal complexes’ spectra, indicating the formation of the complexes. These bands are assigned to π-π* transitions of benzene rings, and the last two bands at lower energy are attributed to a n-π* of azomethine and n-π* of thione form^52^. On complexation, the appearance of new broad bands for Sn and Fe complexes in the visible region between 470 and 491 nm are attributed to ^2^B_2_→^2^A_1_ and ^2^B_2_→^2^E^53^, consistent with a five coordination number, which would have a square pyramidal structure. Similar this result was previously reported^26^. While Zn complexes showed new broad bands in the range 385 and 391 nm due to ^2^B_1_g →^2^A_1_g transition, suggesting that it does not show d-d electronic transition because its d^10^ is completely filled. Thus, it is diamagnetic, and the coordination number is four, and it would have a square planar geometry^54,55^. Generally, complexation with metal ions is used to enhance the fluorescence probe of a ligand, thus increasing some applications of complexes, particularly in the photochemical field. The fluorescence spectra of the TCH ligands and their Sn, Zn, and Fe complexes are shown in Fig. 8(a) and (b). The free TCH ligands, L1 and L2, showed very low emission intensity at 425 nm, possibly due to quenching from the transfer of photo-induced electrons^56^. However, after their complexation with Sn, Zn, and Fe ions, the high intensity of broad emission bands was observed at maximum wavelength between 430 and 476 nm, indicating that they emit a yellow and blue fluorescencentcolor. This result suggests that the TCH unit is a sensitive probe for these ions via the fluorescencent turn-on mechanism^57^. A significant Stokes shift was observed of approximately 120, 121, and 80 nm for Sn, Zn, and Fe, respectively. The L1Zn and L2Zn complexes have shown the highest emission intensity, which means that the TCH ligands are highly sensitive to Zn²⁺. However, the combined effects of chelation, C = N isomerization, and suppression of photo-induced electron transfer led to increased fluorescence^58^. All complexes showed bathochromic shifts compared to the free TCH ligands, which is attributable to excimers^59^. Thus, our new system is a potential candidate in biological and environmental systems for detecting these ions, specifically Zn²⁺ ions, with a high sensitivity.

### Thermal analysis (TGA)

**Fig. 9:**
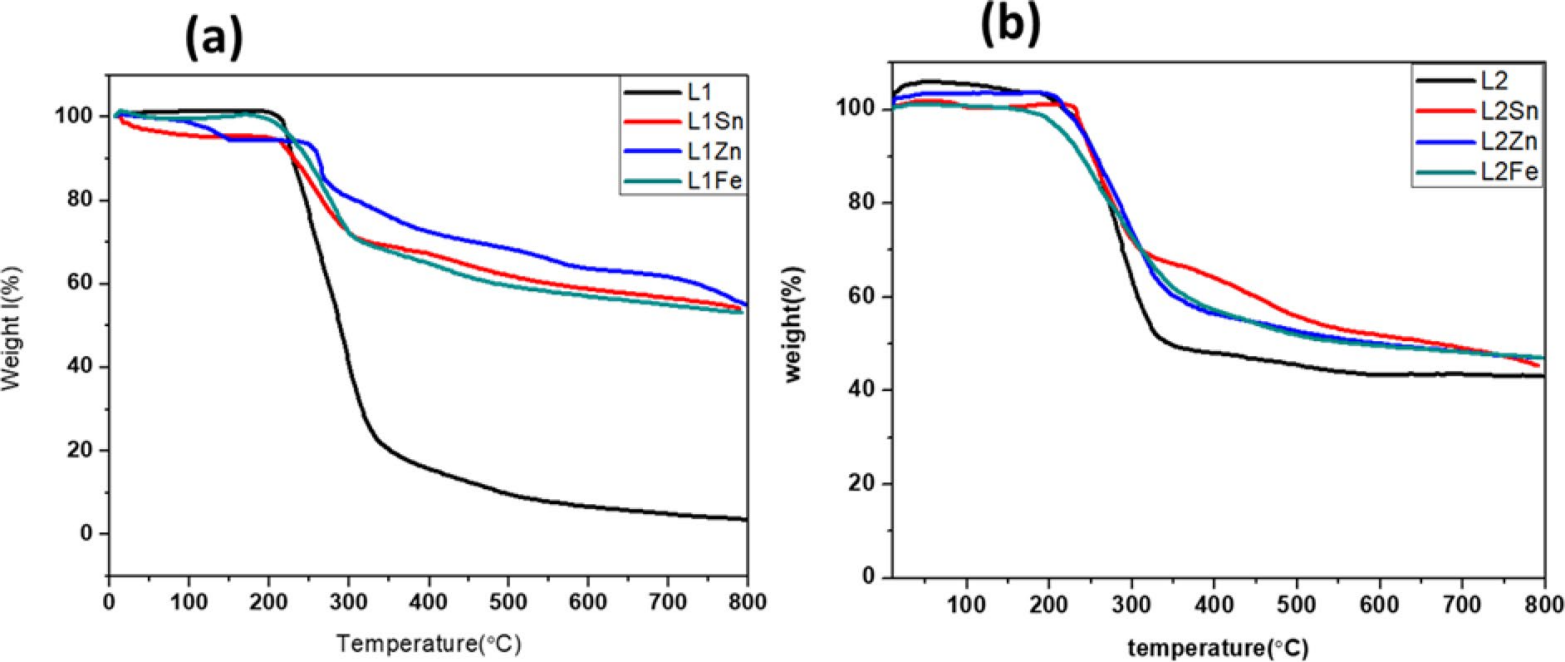
TGA thermograms of (**a**) L1 and (**b**) L2 ligands and their metal complexes.

The thermal behavior and thermal stability of TCH ligands and their metal complexes were investigated using thermal analysis in a nitrogen atmosphere. When the weight loss is plotted as a function of temperature, TGA thermograms were obtained, which are recorded between 25 and 800 °C at a heating rate of 10 °C/min, as shown in Fig. 9(a) and (b). The presence/absence of water molecules and the stages of the complete degradation of the complexes are significant information that can be concluded from the TGA thermogram. It was observed that the weight loss of free L2 is less than that of L1; this may be attributed to its more crystalline nature. In contrast, L1Zn complex exhibited a higher initial decomposition temperature above 300 °C, indicating greater thermal stability. The thermal decomposition behaviors of TCH ligand complexes occur in four stages. The weight loss at 50 to approximately 200 °C is generally due to the loss of lattice and coordinated water molecules. Here, no weight loss was observed corresponding to the release of water molecules, except in the case of the L1Sn complex, which lost 5% of its weight corresponding to the loss of two coordinated water molecules at this temperature range. This result is consistent with the proposed structures for the targeted complexes^60^. The L1Zn complex exhibits a weight loss of 10% within a temperature range of 100–150 °C, attributed to the loss of the solvent molecule that is adsorbed on the surface of the polycrystalline structure. While the second stage involves the loss of terminal methoxy groups and the TCH moiety within the temperature range of 200–300 °C^61^, it is followed by mass loss due to degradation and the removal of the C2H4N2 component, along with other parts of the complexes in the range of 300–500 °C. The residue is observed at temperatures exceeding 500 °C, corresponding to metal oxides and two phenyl groups, without sublimation. All the obtained metal complexes and free L2 exhibited high remaining substances, exceeding 55%, which may be attributed to their crystalline nature, except for the free L1, which aligned with XRD results.

### XRD and SEM studies

**Fig. 10:**
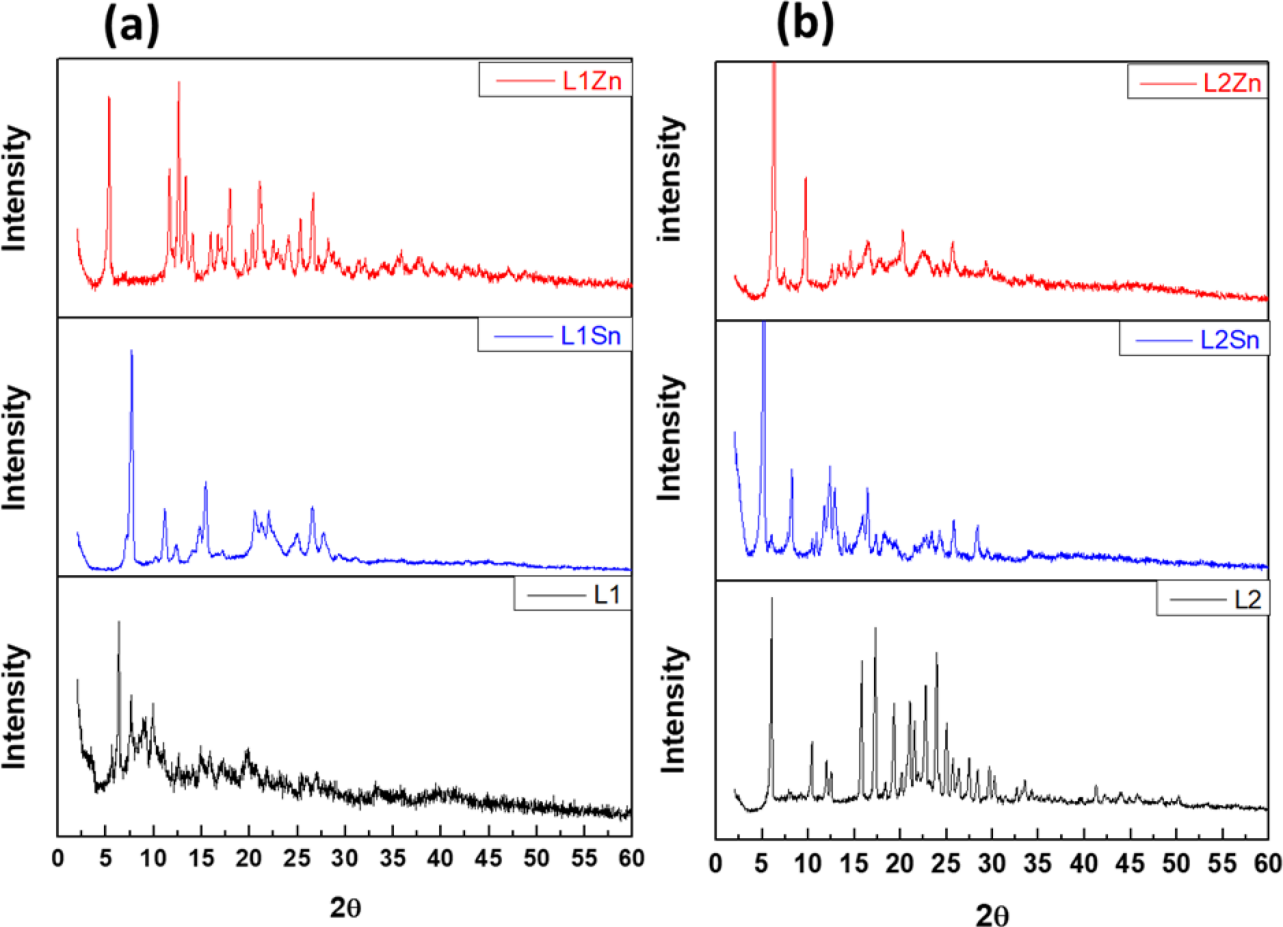
XRD patterns of (**a**) L1 and (**b**) L2 ligands and their metals complexes (Sn and Zn).

**Fig. 11:**
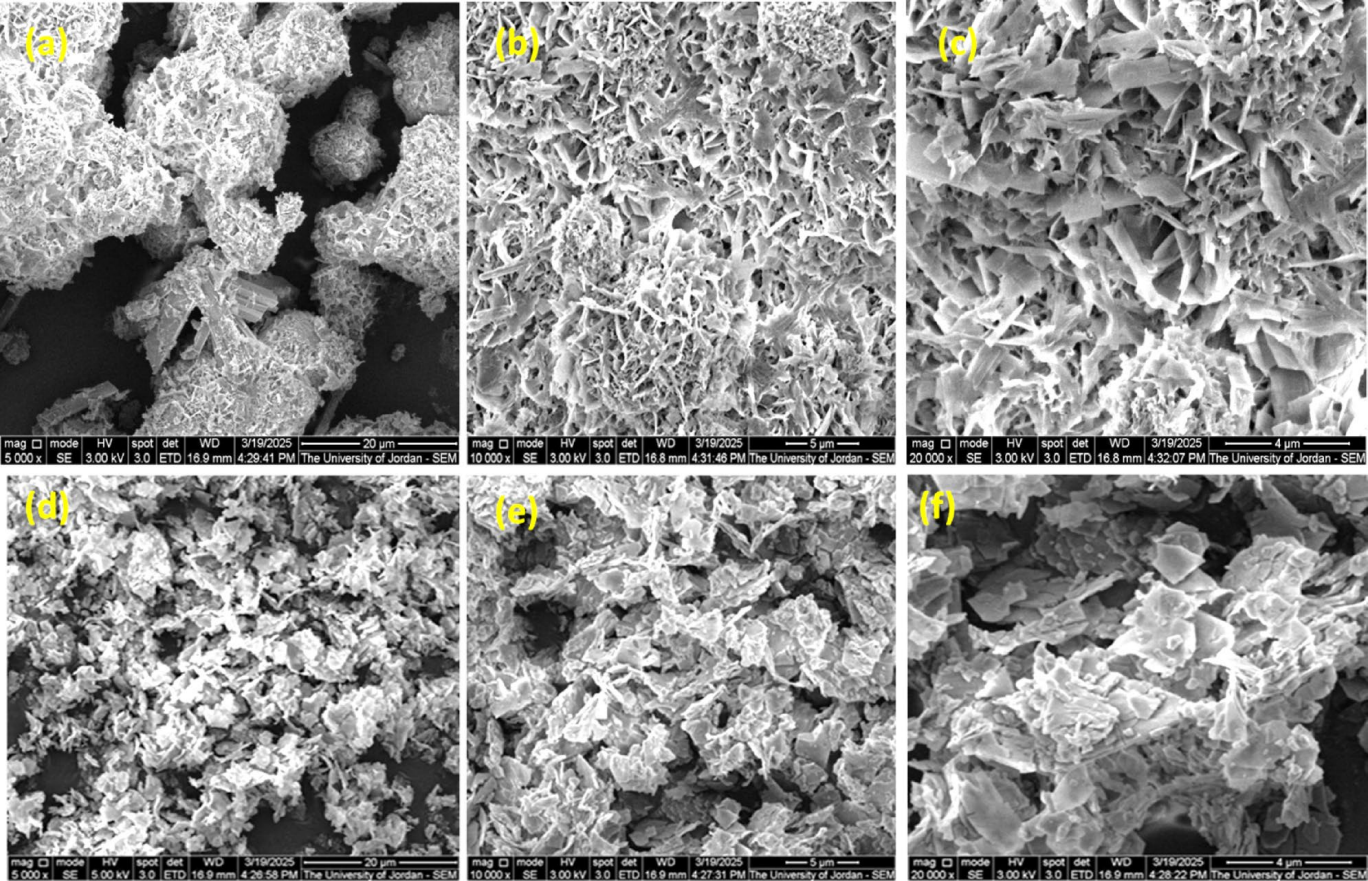
SEM images of L1Zn (**a**–**c**) and L2Sn (**d**–**f**) complexes at different magnifications.

Powder XRD patterns of L1 and L2 and their metal complexes were recorded in the range (2θ = 0 to 60), as shown in Fig. 10(a) and (b). The sharp peaks were observed between 2 theta 5 and 30 °for the TCH ligands, and their metal complexes indicate their crystalline structure. The XRD patterns for metal complexes are completely different from those of the ligands, suggesting the production of novel compounds. The crystallinity of the free TCH ligands, especially L2, may be due to a more symmetrical structure in the case of the presence of methoxy groups in a para position of benzene ring^62^. While, due to the nature of the inherent crystallinity of the metallic compounds, crystallinity appeared in their metal complexes^62^. The Debye–Scherrer’s equation^63^. was used to evaluate the average crystallite size values of the targeted samples, which were foundranging from20 and 50 nm. XRD results revealed the lower grain sizes for these compounds, indicating the formation of nanoparticleswith a polycrystalline and monoclinic crystal structures; consistent with their SEM images^64^ The SEM images obtained for the L1Zn and L2Sn complexes at different magnifications are shown in Fig. 11. The recorded L1Zn SEM images showed a well-developed porous structure and an irregular and heterogeneous surface morphology. The micrographs demonstrate the presence of cracks, fissures, and a few grains of varying sizes in small holes on the external surface of the L1Zn (II). Also, the recorded L2Sn SEM images showed a platelet-like structure. However, our new system has particle sizes in the region of nanoscal in diameter grouped to create larger agglomerates, which can be used in a dye-sensitized solar cell device.

### Biological activity

All the tested compounds showed antimicrobial activity with variable effects against the tested microorganisms. In general, all the compounds had better activity against the Gram-positive bacteria (*S. aureus*) compared to the Gram-negative bacteria and the yeast, with one exception (Table 1). However, this activity is much less than the activity of the two tested commercially available drugs, ciprofloxacin (antibacterial agent) and fluconazole (antifungal agent). It is noteworthy to mention that the complexation with metals did not have a significant effect on the antimicrobial activity of the compounds, except for L2Sn against *C. albicans*, where the activity of the complex was improved by a magnitude of two double dilutions (4 times) compared with its free ligand. Other enhancements in activity can be seen when complexing the L1 with Zn, where the activity was slightly improved by a magnitude of one dilution (from 31.3 µg/mL to 15.6 µg/mL) against *E. coli* and *C. albicans* but not against *S. aureus* (Table 1).

**Table 1:** MIC of the synthetic compounds against *S. aureus*,* E. coli* and *C. albicans*.

Treatment	S. aureus MIC value (µg/mL)	E. coli MIC value (µg/mL)	C. albicans MIC value at 50% inhibition (µg/mL)
L1	15.6	31.3	31.3
L2	15.6	31.3	31.3
L1Sn	15.6	31.3	31.3
L1Zn	15.6	15.6	15.6
L1Fe	15.6	31.3	31.3
L2Sn	15.6	31.3	7.8
L2Zn	15.6	31.3	31.3
L2Fe	15.6	31.3	15.6
Reference drug	Ciprofloxacin 0.097	Ciprofloxacin 0.0008	Fluconazole 3.9

Table 2 presents the mitigation efficacies of both L1 and L2 and their respective metal complexes in LPS-induced inflammation in murine RAW 264.7 macrophages vs. pharmacotherapeutic NSAID (nonsteroidal antiinflammatory drug) indomethacin. Both ligands’ substantial antiinflammation capacities in nanomolarities (nM) were maximally augmented (by respective 2 and 290 fold increase approx.) by Zn complexes (Table 2) outperforming collectively indomethacin’s effects. Their Sn and Fe complexes lacked comparable qualities. Such efficacies were physiologically regulated as evaluated by the lack of relevant cytotoxicities in RAW macrophage incubations. Considerably appreciable DPPH radical scavenging properties of both ligands were marginally enhanced (by 1.5 and 15 folds approx.) by their respective Fe and Zn complexes. Exceptionally L2Sn (but not L1Sn) effected 70fold increase (approx.) in DPPH radical reductive qualities vs. antioxidative ascorbic acid (Table 2). Unlike cisplatin’s unselective cytotoxicities in normal PDL fibroblasts, neither ligands nor metal complexes exerted cytotoxicities undifferentially in the same PDL fibroblast adherent monolayer wells.

**Table 2:** IC_50_ values (nM-µM) of in vitro DPPH-radical scavenging properties vs. ascorbic acid and antiinflammation propensities vs. indomethacin.of the synthetic compounds.

Treatment	DPPH- IC_50_ value (nM- µM)	iNOS- IC_50_ value (nM)	RAW 264.7 viability IC_50_ value	PDL fibroblasts IC_50_ value
L1	250 ± 40 nM***	7.09 ± 0.08 NS	NI	NI
L2	18.07 ± 2.04 µM NS	290 ± 00.0***	NI	NI
L1Sn	6.89 ± 0.76 µM NS	190 ± 20***	NI	NI
L1Zn	25.48 ± 1.69 µM NS	3.67 ± 0.47 NS	NI	NI
L1Fe	180 ± 30 nM NS	300 ± 10***	NI	NI
L2Sn	230 ± 50 nM***	240 ± 40***	NI	NI
L2Zn	12.15 ± 2.05 µM NS	1.04 ± 0.26 NS	NI	NI
L2Fe	1.17 ± 0.24 µM NS	230 ± 40***	NI	NI
Reference drug	Ascorbic acid 0.02 ± 0.01 µM	Indomethacin 30 ± 5 nM	Indomethacin: NI	Cisplatin 5.6 ± 0.53 µM

Lack of cytotoxicity of the same panel of synthetic compounds against normal PDL fibroblasts vs. cisplatin and against RAW264.7 macrophages vs. indomethacin.

Results are mean ± SD (*n* = 4 independent replicates). IC_50_ values (µM) (concentration at which 50% of DPPH reduction or antiinflammation in comparison to non-induced basal incubations) were calculated within testing dose range (1.563 or 6.25–200µM).

* DPPH* diphenyl-2-picryl-hydrazyl,* iNOS* induced nitric oxide synthase,* NI* non-inhibitory over the tested dose ranges.

Significance level of *P < 0.05 and ***P < 0.001 vs. cisplatin; the Positive control.* NS* not significantly different from respective cisplatin’s IC_50_ values.

Tables 3, 4 and 5 demonstrate that cisplatin, the robust apoptogenic antineoplastic agent, effected exceptional antiproliferation propensities in adherent monolayers of all adenocarcinomas of colorectal and pancreatic (Table 3), skin, lung, prostate, uterine cervix, and glioblastoma (Table 4), as well as all 5 mammary tumor cell lines (Table 5).Nevertheless, neither ligands nor any of their metal complexes proved efficacious in viability reductions of cancerous pancreatic PANC1 (Table 3), prostate PC3 (Table 4), or any of the mammary adenocarcinomas of MCF7, or invasive malignancies of MDA-MB-453 and BT549 (Table 5). Tables 3 and 4 illustrate that L1 (unlike L2) had substantially pronounced cytotoxicity in CACO2, SW480, SW620, and glioblastomas but less appreciable viability reductions in HCT116 and HT29. Also, highly marked growth inhibitions were found for both Zn and Fe complexes of L1 in colorectal cancer incubations in all HCT116, HT29, CACO2, SW480, and SW620. Comparably, L1Sn complex (but not its L1 complex) was found to have significant antiproliferation capacities in HCT116, SW480, and SW620. Impressively substantial viability reductions of L2Zn complexes in both colorectal cancer SW480 and SW620 and of L2Fe complexes in SW620 were notable (Table 3). Similarly, Sn and Zn complexes for L1 (unlike their free L1) were substantially cytotoxic in skin melanoma incubations. Furthermore, L1Zn (but none of L1Fe complexes) was superbly antiproliferative in cancer adherent monolayers of lung, uterine cervix, and glioblastoma (Table 4). L2Sn had marginally or minimally antiproliferation efficacies in colorectal cancer adherent monolayers (Table 3), skin, lung, prostate, uterine cervix, or glioblastoma (Table 4). The L2Fe complex (unlike its free L2) was found to have marked cytotoxicity in lung tumor cells. Noticeably in Table 5 of both mammary adenocarcinomas of T47D and aggressive MDA-MB-231 adherent monolayers, comparable antiproliferative capacities were exerted by L1 and its both Zn and Fe complexes, as well as L2Zn complex, unlike its marginally effective ligand alone or any of its other metal complexes.

**Table 3:** Cytotoxicity (as of %control) IC_50_ value in µM of the synthetic compounds vs. cisplatin against invasive pancreatic and colorectal malignancy cell lines.

Treatment	HCT116	HT29	CACO2	SW480	SW620	PANC1
L1	36.1 ± 1.9***	42.1 ± 5.7**	23.2 ± 0.6 NS	5.35 ± 0.25**	2.19 ± 0.22 NS	66.8 ± 0.2***
L2	506.2 ± 9.5***	293.8 ± 23.4***	462.3 ± 5.1***	51.57 ± 3.07***	117.10 ± 3.81***	263.0 ± 11.0***
L1Sn	28.4 ± 4.8***	70.0 ± 0.4***	57.5 ± 0.2***	9.88 ± 0.91 NS	14.96 ± 1.68*	325.6 ± 1.9***
L1Zn	21.0 ± 0.6*	23.1 ± 0.0 NS	22.8 ± 0.3 NS	2.12 ± 0.31***	1.94 ± 0.25 NS	47.4 ± 4.0 NS
L1Fe	13.2 ± 0.6 NS	16.7 ± 0.1 NS	14.2 ± 1.7 NS	12.18 ± 0.74*	0.90 ± 0.14 NS	74.5 ± 3.8***
L2Sn	375.4 ± 19.4***	214.4 ± 32.0***	462.4 ± 36.4***	52.78 ± 0.69***	290.44 ± 13.10***	423.7 ± 3.2***
L2Zn	84.9 ± 6.2***	29.9 ± 0.6 NS	59.8 ± 1.1***	28.10 ± 0.85***	8.15 ± 1.63 NS	127.8 ± 4.2v***
L2Fe	127.4 ± 0.2***	58.2 ± 3.1***	84.9 ± 1.3***	17.65 ± 0.21***	98.46 ± 5.71***	287.1 ± 5.8***
Cisplatin	0.05 ± 0.01	0.8 ± 0.1	4.4 ± 0.2	8.92 ± 0.24	0.32 ± 0.04	41.5 ± 0.4

Results are mean ± SD (*n* = 4 independent replicates). IC_50_ values (concentration at which 50% inhibition of cell proliferation took place in comparison to non-induced basal 72 h incubations) were calculated within 3.125-200µM range. Significance level of *P < 0.05 and ***P < 0.001 vs.. cisplatin; the Positive control.* NS* not significantly different from respective cisplatin’s IC_50_ values.

**Table 4:** Cytotoxicity (as of %Control) IC_50_ value in µM of the synthetic compounds vs. cisplatin against other cancer cell lines (skin, lung, prostate, uterine cervix and glioblastoma).

Treatment	A375 skin melanoma	A549 lung malignancy	PC3 prostate malignancy	Hela cervix malignancy	U87 Gliobalstoma
L1	41.7 ± 0.7***	44.8 ± 1.8***	144.7 ± 1.5 NS	44.8 ± 3.8 NS	29.5 ± 0.2 NS
L2	362.3 ± 9.9***	36.6 ± 0.2***	1024.7 ± 168.2***	374.0 ± 3.1***	20756.4 ± 3753.4***
L1Sn	16.5 ± 1.7 NS	83.5 ± 1.3***	140.6 ± 5.9 NS	2242.0 ± 90.9***	4855.2 ± 178.0**
L1Zn	10.5 ± 1.4 NS	13.9 ± 0.0***	62.1 ± 1.8 NS	17.8 ± 2.6 NS	14.6 ± 0.7 NS
L1Fe	47.7 ± 3.9***	46.9 ± 0.6***	234.9 ± 26.4**	45.1 ± 0.6 NS	41.2 ± 0.1 NS
L2Sn	125.4 ± 4.0***	102.3 ± 2.5***	840.3 ± 13.8***	137.9 ± 11.0**	99.7 ± 1.0 NS
L2Zn	95.6 ± 0.1***	85.7 ± 0.7***	251.7 ± 6.8***	100.9 ± 0.9*	853.7 ± 119.1 NS
L2Fe	104.8 ± 3.7***	19.3 ± 1.0***	210.9 ± 29.0**	288.0 ± 1.6***	1000.2 ± 99.0 NS
Cisplatin	10.5 ± 1.4	71.9 ± 2.9	12.5 ± 0.2	27.3 ± 0.9	14.6 ± 2.3

Results are mean ± SD (*n* = 4 independent replicates). IC_50_ values (concentration at which 50% inhibition of cell proliferation took place in comparison to non-induced basal 72 h incubations) were calculated within 3.125-200µM range Significance level of *P < 0.05 and ***P < 0.01 − 0.001 vs.. cisplatin; the Positive control.* NS* not significantly different from respective cisplatin’s IC_50_ values.

**Table 5:** Cytotoxicity (as of %control) IC_50_ value in µM of the synthetic compounds vs. Cisplatin against mammary malignancies cell lines.

Treatment	MCF7 mammary malignancy	T47D mammary malignancy	MDA-MB-231 invasive mammary malignancy	MDA-MB-453 invasive mammary malignancy	BT-549 invasive mammary malignancy
L1	52.43 ± 5.25 NS	21.8 ± 1.2***	29.2 ± 3.8**	72.9 ± 3.4 NS	46.37 ± 1.11*
L2	232.38 ± 37.1***	205.0 ± 4.9***	228.0 ± 12.7***	935.3 ± 163.0***	94.84 ± 4.17***
L1Sn	263.50 ± 38.89***	35.4 ± 1.6 NS	55.4 ± 4.3***	72.5 ± 0.4 NS	54.82 ± 1.63*
L1Zn	47.69 ± 3.32 NS	23.3 ± 0.6***	25.1 ± 0.8*	98.2 ± 2.3 NS	34.04 ± 4.02 NS
L1Fe	183.20 ± 6.72***	9.0 ± 1.1***	11.0 ± 2.6 NS	36.9 ± 2.4 NS	217.75 ± 31.15***
L2Sn	434.64 ± 35.44***	128.9 ± 1.9***	152.7 ± 3.2***	661.7 ± 127.9***	225.78 ± 37.90***
L2Zn	157.17 ± 7.74***	25.1 ± 0.0***	27.7 ± 0.1*	151.9 ± 2.8 NS	97.66 ± 4.19***
L2Fe	135.06 ± 6.84***	69.0 ± 1.2***	101.8 ± 6.1***	205.1 ± 21.8*	154.40 ± 13.51***
Cisplatin	31.59 ± 0.44	34.2 ± 0.5	11.8 ± 1.4	9.8 ± 0.1	3.77 ± 0.25

Results are mean ± SD (*n* = 4 independent replicates). IC_50_ values (concentration at which 50% inhibition of cell proliferation took place in comparison to non-induced basal 72 h incubations) were calculated within 3.125-200 µM range. Significance level of *P < 0.05 vs.. cisplatin; the Positive control. * NS* not significantly different from respective cisplatin’s IC_50_ values.

### Structure-activity relationship (SAR)

The L1 compound with iron has demonstrated excellent activity primarily against colorectal cancer cell lines. Meanwhile, the L1 compound with zinc has also shown promising IC_50_ values, although it is less active than its iron counterpart. It is suggested that the presence of ortho-methoxy facilitates the formation of a trivalent coordination bond with iron. This bond enables a parallel trivalent coordination with the receptor, similar to what occurs with topoisomerase II in colorectal cancer. This phenomenon was also discussed in our previous research on the functionality of iron-trivalent fluoroquinolone compounds, particularly concerning colorectal cancer cell lines^65^.

**Scheme 1:**
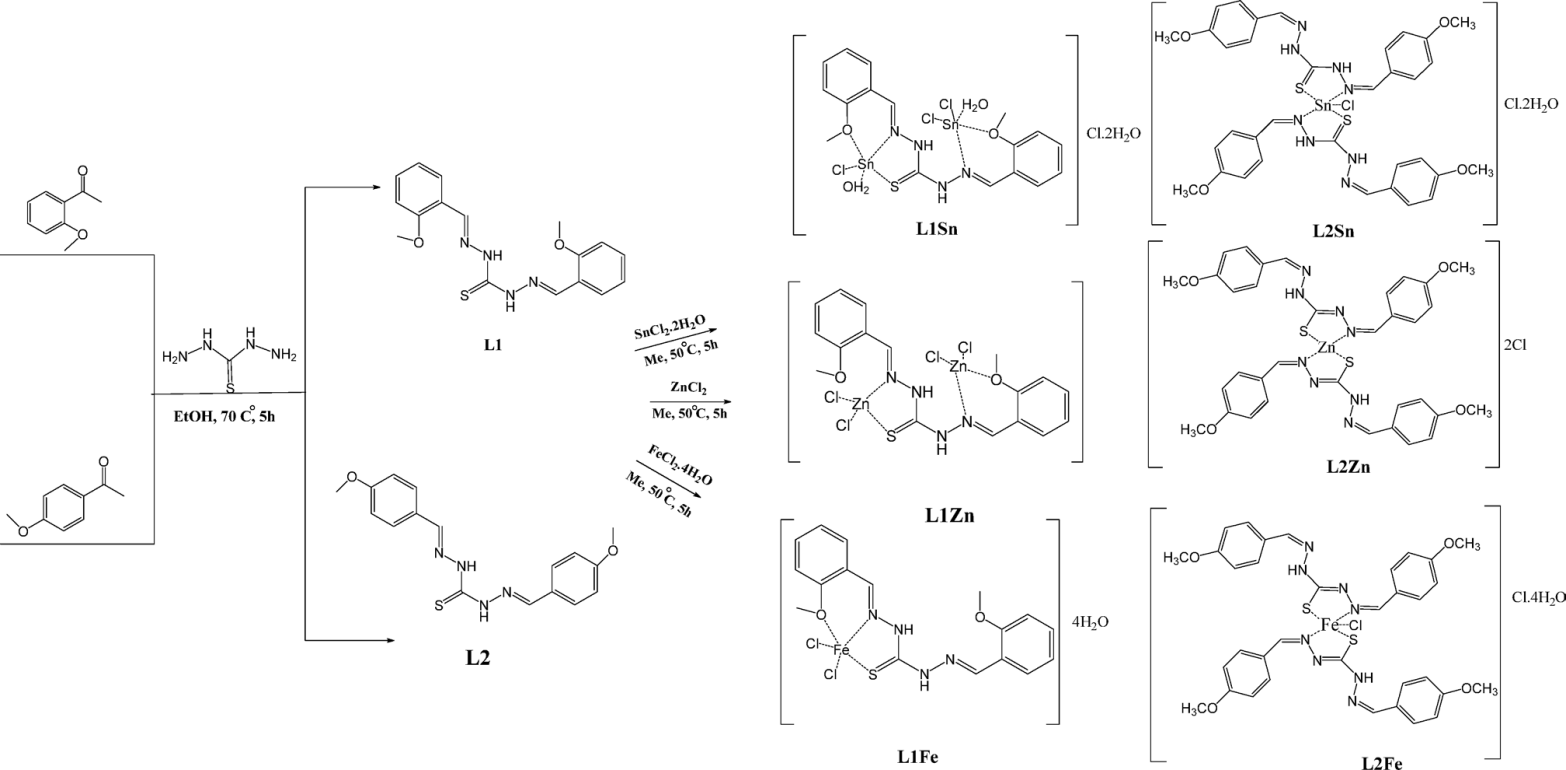
Representative the synthesis rote of TCH ligands and their Sn, Zn, and Fe complexes with their proposed structures.

## Concluding remarks and future directives

This research effectively coordinated two novel Schiff bases derived from the TCH core, which exhibit bidentate (NS) and tridentate (NSO) donor sites, with Sn, Zn, and Fe metals. The proposed structures of the resulting complexes were thoroughly determined. On complexation, an enhancement of the fluorescence emission of the resulting complexes was observed. This may be attributed to a potent chelation-enhanced fluorescence effect, indicating their potential use in developing fluorogenic sensors for Sn^2+^, Zn^2+^, and Fe^2+^. The findings from Scanning Electron Microscopy and X-ray Diffraction demonstrated that Zn and Sn complexes and the free L2 displayed a nano-crystalline structure, with average crystallite sizes ranging from 20 nm to 50 nm, suggesting their usefulness as precursors for nanoparticle synthesis. All compounds demonstrated variable antimicrobial activities against *Staphylococcus aureus*, *Escherichia coli*, and *Candida albicans*, although their effectiveness was lower than commercially available drugs. Both L1Fe and L1Zn proved as most promising complexes against colorectal cancer cells. Of primary novelty in adherent monolayers of normal PDL fibroblasts, and unlike cisplatin, neither ligands nor any of their metal complexes exerted un-differential cytotoxicities. Mechanistically, in LPS induced inflammation in macrophages; L1 and L2’s remarkable antiinflammation in nanomolarities (nM) were maximally augmented by Zn complexes (but not their Sn or Fe complexes), thus outperforming indomethacin and without cytotoxicities in macrophage incubations. Exceptionally L2Sn effected optimal enhancement and strongest biological activity in DPPH radical scavenging activity vs. the well-known antioxidant, ascorbic acid. Taken together, the utility of metal complexes of ligands were optimally signified via utmost maximal antiinflammation and anitioxidation capacities exceeding those of respective reference agents; hence forth these complexes have a promising potential to be used as fluorescence chemosensors in the diagnosis and monitoring of cancer cells in addition to their ability to treat some types of cancer i.e. as theranostic agents (diagnostic and therapeutic agent) and to treat some inflammatory disorders.

## Electronic supplementary material

Below is the link to the electronic supplementary material.

Supplementary Material 1

## Data Availability

All data generated or analyzed during this study are available from the corresponding author upon reasonable request.
