# Supplementary material for: Multi-biological activity evaluation of Sn(П), Zn(П) and Fe(П) complexes based on thiocarbohydrazide schiff bases: synthesis, spectroscopic investigations and fluorescence studies
Source: Sci Rep. 2025 Aug 28;15:31757. doi: 10.1038/s41598-025-09239-w (PMC12394552; doi:10.1038/s41598-025-09239-w)
Supplement: Supplementary file 1 — Supplementary Material 1 [file 41598_2025_9239_MOESM1_ESM.docx]

**Multi-biological Activity Evaluation of Sn(П), Zn(П) and Fe(П) Complexes Based on Thiocarbohydrazide Schiff Bases: Synthesis, Spectroscopic Investigations and Fluorescence Studies**

Abdulsalam Mahdy^a,b^*, Jalal A. Zahra^a^ , Randa N. Haddadin^c^, Yusuf Al-Hiari^d^, Violet Kasabri^e^*

^a^ Chemistry Department, School of Science, The University of Jordan, Amman, Jordan.

^b^ Chemistry Department, Faculty of Education & Science - Rada'a, Albaydha University, Albaydha, Yemen.

^c^ Department of Pharmaceutics & Pharmaceutical Technology, School of Pharmacy, The University of Jordan.

^d^Department of Pharmaceutical Sciences, School of Pharmacy, The University of Jordan, Amman, Jordan ^e^Department of Biopharmaceutics and Clinical Pharmacy, School of Pharmacy, The University of Jordan, Amman, 11942, Jordan

**Preparation of TCH from hydrazine and carbon disulfide**

TCH was prepared by the procedure reported in the literature.[27] The required weight of hydrazine hydrate (0.4 mole, 20g) was put into a 500 ml two necks round bottomed flask that connected to a reflux apparatus and stirred at room temperature. Then 0.08 moles, 6g of carbon disulfide was added slowly with stirring for 1h. After that, 120 ml of methanol was added to the reaction mixture and the temperature was raised to 60 °C for 30 min. The progress of the reaction was tracked using a TLC technique (95% chloroform: methanol). After end of the reaction (3h), the system was cooled to room temperature and the precipitate was filtered. The resultant precipitate, TCH, was washed with methanol and dried. After the recrystallization process was done in warm water, the white needle crystals was obtained and washed with methanol and dried in vacuum oven, yielding, 95 %. ^1^H-NMR (500 Hz, DMSO, δ, ppm) 8.6 (s, 6H, NH and NH_2_) and ^13^C-NMR (500 Hz, DMSO, δ, ppm) 182.2(s, 1C, C=S, cf. Figure S1)


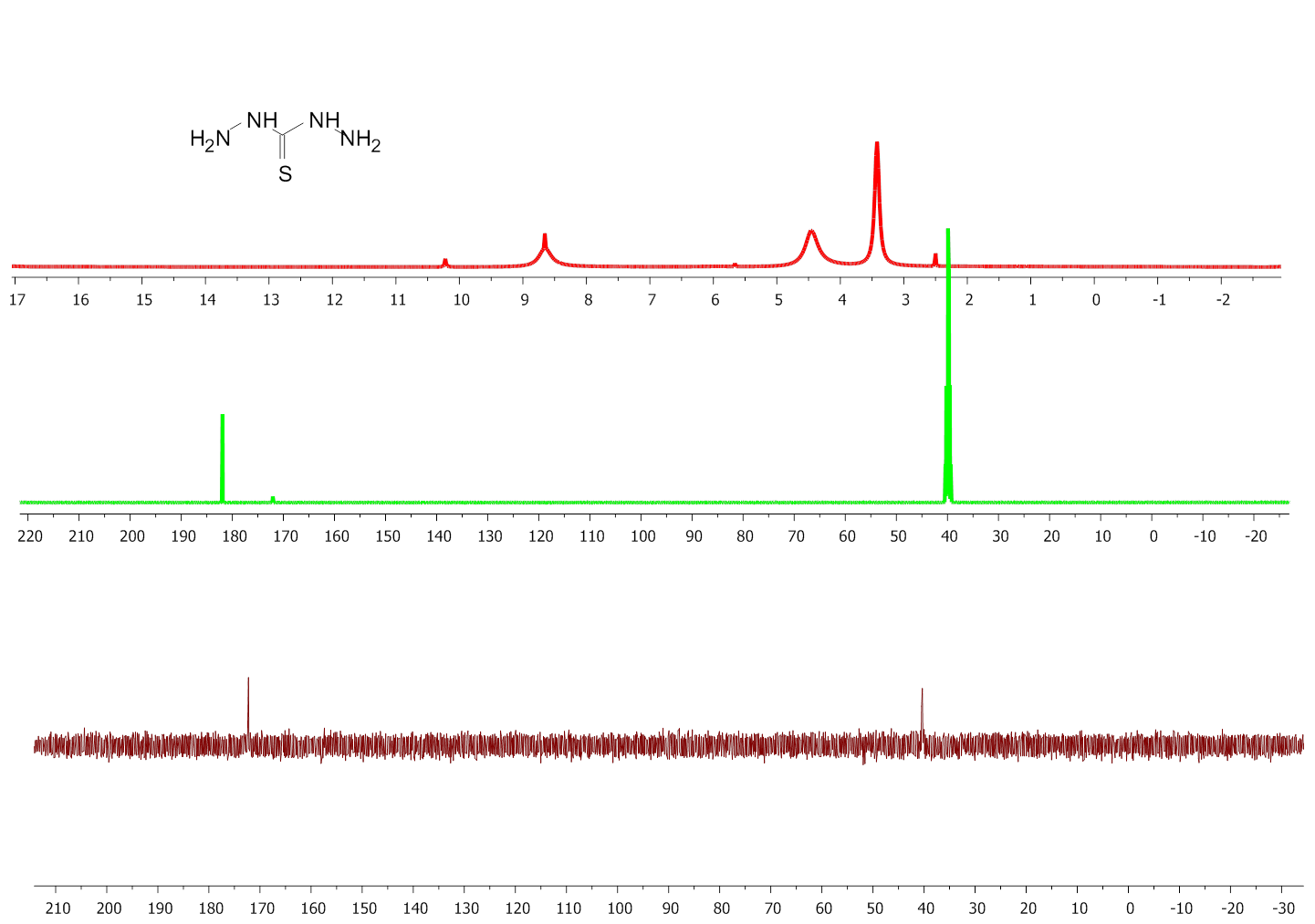


Figure S1: NMR (^1^H, ^13^C and DEPT-135) of Thiocarbohydrazide (TCH).


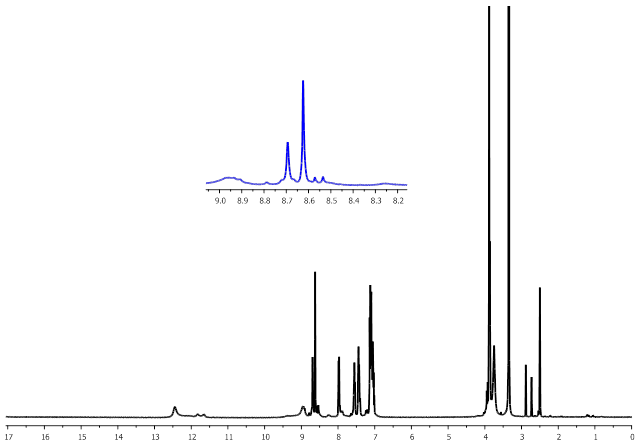


Figure S2: ^1^H-NMR of L1Sn

Figure S3: ^1^H-NMR of L1Zn


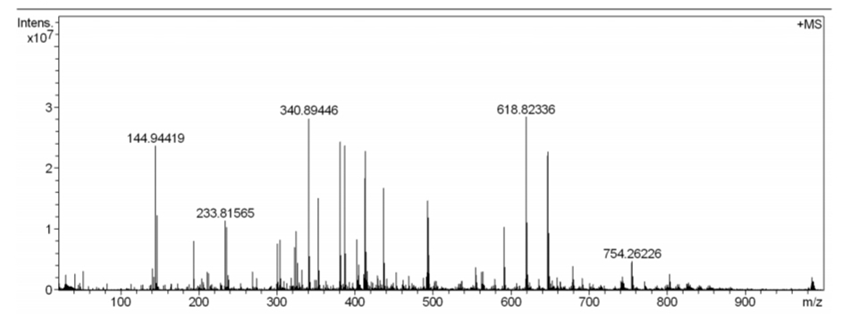


Figure S4: Mass spectrum of L1Zn


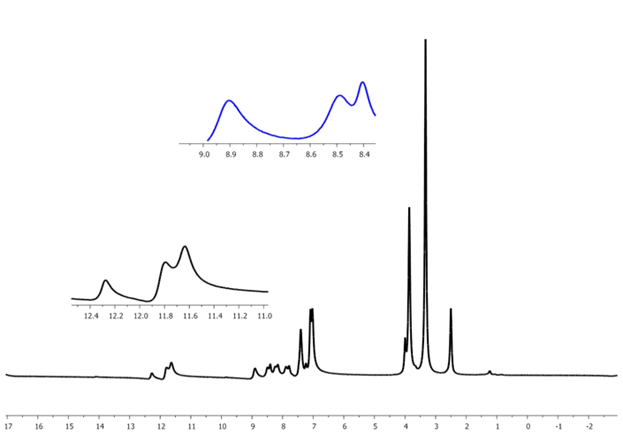


Figure S5: ^1^H-NMR of L1Fe


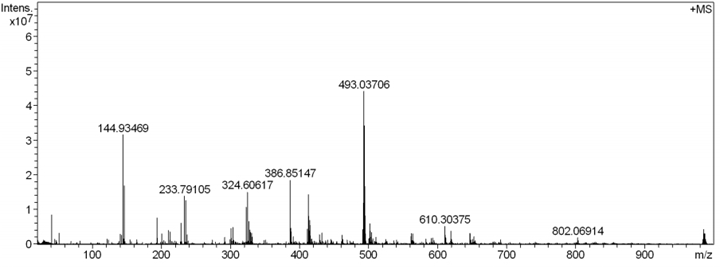


Figure S6: Mass spectrum of L1Fe


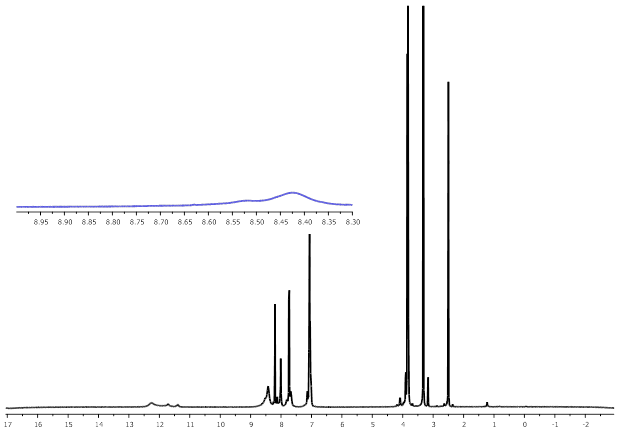


Figure S7: ^1^HNMR of L2Sn


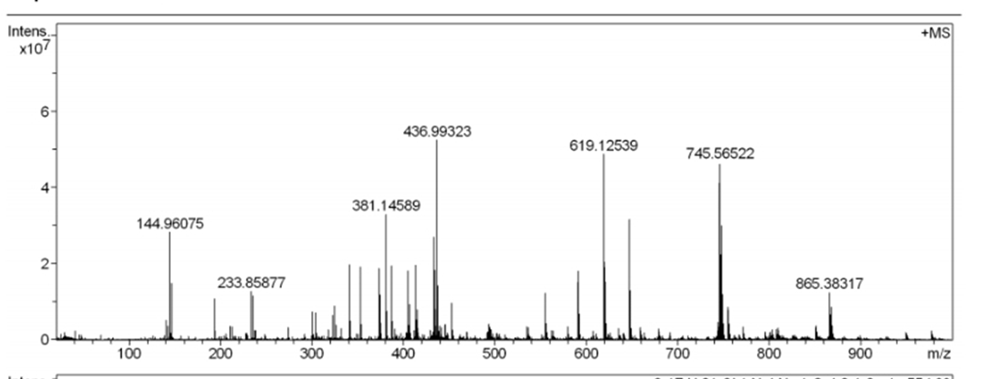


Figure S8: Mass spectrum of L2Sn


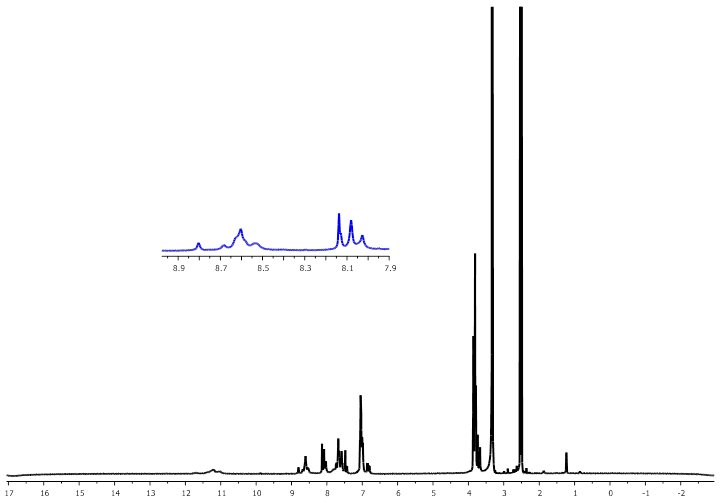


Figure S9: ^1^H-NMR of L2Zn


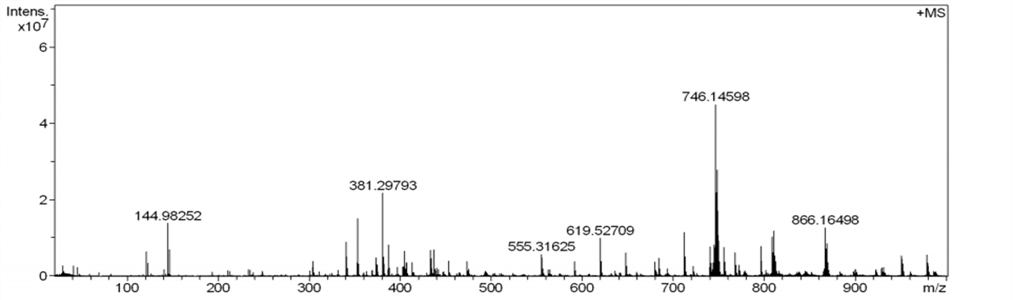


Figure S10: Mass spectrum of L2Zn

Figure S11: ^1^H-NMR of L2Fe


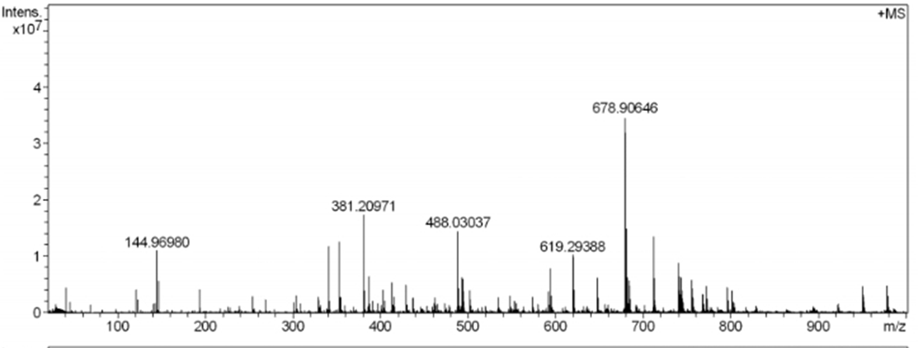


Figure S12: Mass spectrum of L2Fe

Table S1: physical properties and analytical results of TCH ligands and their metal complexes

| Compound | Chemical formula | Yield (%) | Color | M.P. °C | Mass spectra | |
| --- | --- | --- | --- | --- | --- | --- |
|  |  |  |  |  | Calc. | found |
| L1 | C17H18N4O2S | 95 | Cream | 185-190 | 381.12 | 381.0839 |
| L2 | C17H18N4O2S | 95 | white | 180-185 | 381.12 | 381.0804 |
| L1Sn | C17H22Cl3N4O4SSn2 | 85 | Yellow | 195-200 | 745.84 | 745.81 |
| L1Zn | C17H16Cl4N4O2SZn2 | 60 | Ivory | 260-265 | 648.80 | 648.79 |
| L1Fe | C17H23ClFeN4O4S | 85 | brown | 150-155 | 492.17 | 493.03 |
| L2Sn | C34H36ClN8O4S2Sn | 80 | yellow | 212-215 | 862.12 | 865.48 |
| L2Zn | C34H34N8O4S2Zn | 70 | Ivory | 166-170 | 746.14 | 746.15 |
| L2Fe | C34H34ClFeN8O4S2 | 84 | brown | 162-165 | 796.12 | 796.11 |

Table S2: FTIR results of TCH ligands and their metals complexes

| Compound | IR spectra (cm^-1^) | | | | | | |
| --- | --- | --- | --- | --- | --- | --- | --- |
|  | N-H | C=N | NH-C=S | C-H  (O-CH_3_) | C=S | M-N | M-S |
| L1 | 3271, 3101 | 1606 | 1387 | 2837 | 755 |  |  |
| L2 | 3271, 3142 | 1609 | 1387 | 2849 | 833 |  |  |
| L1Sn | 3252 | 1643 | 1387 | 2852 | 742 | 592 | 682 |
| L1Zn | 3255 | 1636 | 1385 | 2835 | 752 | 482 | 543 |
| L1Fe | 3224 | 1635 | 1385 | 2835 | 738 | 572 | 678 |
| L2Sn | 3256 | 1641 | 1387 | 2843 | 817 | 506 | 670 |
| L2Zn | 3240 | 1638 | 1384 | 2842 | 814 | 515 | 672 |
| L2Fe | 3240 | 1638 | 1384 | 2845 | 814 | 515 | 644 |

Table S3: UV and molar conductivity results of TCH ligands and their metal complexes

| Compound | ʎ_max_ | Assigned | Molar conductivity (µs/cm) | geometry |
| --- | --- | --- | --- | --- |
| L1 | 277, 344, 353 | п-π*, n-π* and n-π* |  |  |
| L2 | 260, 338, 357 | п-π*, n-π* and n-π* |  |  |
| L1Sn | 253, 337, 366, 396, 491 | п-π*, n-π*, n-π*, and ^2^B_2_ → ^2^A_1_  ^2^B_2_ → ^2^E | 76.5 | square pyramidal |
| L1Zn | 262, 336, 365, 391 | п-π*, n-π* , n-π* and ^2^B_1_g → ^2^A_1_g | 8.7 | Square planar |
| L1Fe | 266, 333, 357, 480 | п-π*, n-π* , n-π* and, ^2^B_2_ → ^2^E | 70.4 | square pyramidal |
| L2Sn | 260, 320, 364, 391, 492 | п-π*, n-π* , n-π* and ^2^B_2_ → ^2^A_1_  ^2^B_2_ → ^2^E | 71.8 | square pyramidal |
| L2Zn | 272, 324, 360, 385, | п-π*, n-π* , n-π* and ^2^B_1_g → ^2^A_1_g | 3.6 | Square planar |
| L2Fe | 282, 347, 361, 470 | п-π*, n-π* , n-π* and ^2^B_2_ → ^2^E | 5.6 | square pyramidal |
